# Rye Straw Lignin as a Promising Source of Tricin: Varietal Differences and Its Release Using Deep Eutectic Solvents

**DOI:** 10.1021/acssuschemeng.6c02660

**Published:** 2026-04-10

**Authors:** Javier Benito, Raquel Cañadas, Francisco Barro, Ana Gutiérrez, André M. da Costa Lopes, Nalin Seixas, Sonia A. O. Santos, Armando J. D. Silvestre, José C. del Río, Jorge Rencoret

**Affiliations:** † Instituto de Recursos Naturales y Agrobiología de Sevilla, CSIC, Reina Mercedes 10, Seville 41012, Spain; ‡ Instituto de Agricultura Sostenible (IAS), CSIC, Av. Menéndez Pidal, S/N, Córdoba 14004, Spain; § CICECO-Aveiro Institute of Materials and Department of Chemistry, 426216 University of Aveiro, Campus de Santiago, Aveiro 3810-193, Portugal

**Keywords:** agricultural residue, 2D-NMR, tricin, valorization, deep eutectic solvents, DES

## Abstract

Lignins isolated from the straw of six rye (*Secale cereale*) varieties (Cl 98, Slapske, Zidlochovicke Panis, Ceske Normalni, Bates, and SU Stakkato) were comprehensively characterized by advanced analytical techniques to assess their structural features and valorization potential. Rye straws contained ∼15% of lignin, which was primarily constituted by guaiacyl (∼52–61%) and syringyl (∼35–45%) units, with minor amounts of *p*-hydroxyphenyl units (∼3%). The lignin backbone was enriched in β–*O*–4′ alkyl–aryl ether linkages (77–79%), followed by β–5′ (10–12%) and β–β′ (4–6%). All the lignins from the rye straws were enriched in tricin, with the Cl 98 variety having the highest content, reaching up to 26.2 g/kg straw. Since tricin is incorporated exclusively through 4′–*O*–β ether linkages, an environmentally benign deep eutectic solvent (DES, choline chloride/lactic acid, 1:10), which is known to efficiently cleave such bonds, was evaluated for its selective release. DES treatment of Cl 98 straw yielded 1.92 mg of free tricin/g straw, confirming the effective cleavage of β–*O*–4 linkages. This study highlights rye straw lignin as a tricin-enriched, renewable biopolymer, emphasizing its potential as a sustainable source for high-value tricin recovery and supporting the valorization of cereal crop residues within lignocellulosic biorefineries.

## Graphical abstract

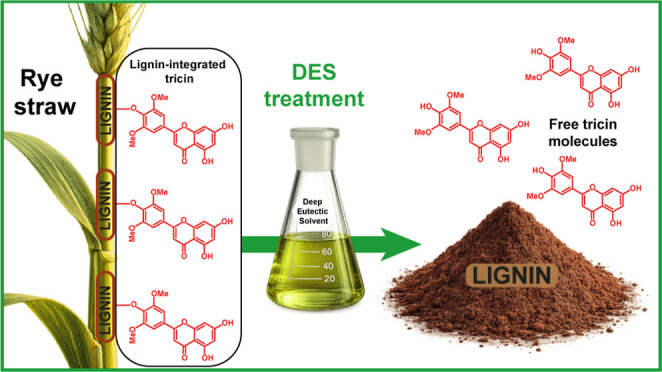

## 1 Introduction

The transition toward a circular bioeconomy has intensified the search for sustainable and renewable biomass sources for the production of fuels, chemicals, and materials.^1,2^ Agricultural residues, such as cereal straws, are abundant lignocellulosic feedstocks that are often underutilized and present significant potential for valorization within the biorefinery frameworks.^3^ Among these, rye (*Secale cereale L*) straw is gaining increasing attention due to its robust growth under marginal conditions, its high biomass productivity, and wide availability.^4^

Rye is among the most widely cultivated cereals worldwide, grown predominantly in temperate regions of Europe, Russia, and North America. According to the Food and Agriculture Organization (FAO), in 2024, the global rye grain production exceeds 11.5 million tons.^5^ The corresponding rye straw biomass, typically amounting to 1.3–1.6 times the grain yield,^6^ represents a substantial and renewable lignocellulosic resource. Despite this availability, rye straw is frequently treated as waste or used for low-value purposes such as animal bedding or soil cover. Given its considerable content of cellulose (∼31%), hemicelluloses (∼22%), and lignin (∼25%),^7^ this material represents an attractive feedstock for integrated biorefinery processes aimed at producing both biofuels and high-value chemicals and materials. While the polysaccharide fraction of rye straw has been widely examined for its potential to yield fermentable sugars,^7^ its lignin fraction remains comparatively understudied, even though lignin is both a key structural polymer and a renewable source of aromatic compounds.^8^

Lignins from gramineous (grass-derived) species, including rye, differ from those of hardwoods and softwoods in that they incorporate, in addition to the conventional monolignols (*p*-coumaryl, coniferyl, and sinapyl alcohols), also significant amounts of *p*-hydroxycinnamates (ferulates and *p*-coumarates),^9^ as well as the flavonoid tricin.^10–15^ In particular, tricin has attracted considerable interest due to its antioxidant, anti-inflammatory, and anticancer properties,^16–19^ positioning it as a high-value coproduct in lignocellulosic biorefineries. In grasses, tricin participates directly in lignin biosynthesis through forming 4′–*O*–β ether linkages with monolignols, thereby acting as a lignin chain initiator.^10,14^ Its integration into the lignin macromolecular structure by relatively labile ether linkages offers a unique opportunity for its recovery under mild depolymerization conditions.

Among the most promising approaches to enable such ether bond cleavage is the use of deep eutectic solvents (DES), a class of neoteric solvents formed by at least one hydrogen-bond acceptor (HBA) and one hydrogen-bond donor (HBD), that have emerged as versatile, greener media for lignocellulosic fractionation. For instance, choline chloride/lactic acid (ChCl/LA) at molar ratio 1:10 is a reference combination of HBA and HBD, that has been successfully tested in literature.^20^ ChCl and LA bring the increased advantage of being both from natural origin, which increases the overall green connotation of these media. On the other hand, the use of acidic DES formulations, such as ChCl/LA (1:10) can promote solvolytic/acidolytic cleavage of aryl–ether linkages (especially β–ether bonds),^21^ offering an attractive platform for the release of lignin-bound phenolics and high-value flavonoids such as tricin under comparatively mild conditions.

However, despite the potential of the rye straw as source of tricin, comprehensive studies on the lignin composition and tricin content across different rye varieties are scarce. Elucidating varietal differences is essential for optimizing rye straw valorization in integrated biorefineries, particularly for the coproduction of high-value lignin-derived molecules such as tricin.

In this study, we conducted a comprehensive chemical and structural characterization of lignins isolated from the straws of six genetically distinct rye (*S. cereale L*.) varieties. In this context, native-like lignins were isolated using established methods,^22^ and subsequently analyzed using a suite of advanced analytical techniques, including pyrolysis–gas chromatography/mass spectrometry (Py-GC/MS), nuclear magnetic resonance (NMR), gel permeation chromatography (GPC), and derivatization followed by reductive cleavage (DFRC), to elucidate lignin monomer composition, interunit linkages, acylation patterns, and tricin incorporation. Additionally, and taking into account the potential of DES to induce lignin extraction,^20,21^ the rye variety exhibiting the highest tricin content was subjected to an exploratory delignification treatment with the ChCl/LA (1:10) to assess the potential release of tricin in its native form. The findings provide new insights into the structural diversity of rye straw lignin and emphasize its potential as a renewable source of bioactive flavonoids and aromatic building blocks, supporting the advancement of integrated lignocellulosic biorefineries.

## 2 Experimental Section

### 2.1 Chemicals

All chemicals were purchased from Sigma-Aldrich (Germany), except NHND (Alfa Aesar), and were used without further purification unless otherwise stated. The chemicals included: dimethyl sulfoxide-*d*_6_ (99.9% D), tetramethylammonium hydroxide (25 wt % in methanol), 2-chloro-4,4,5,5-tetramethyl-1,3,2-dioxaphospholane (95%), choline chloride (≥98%), lactic acid (>80%), ethanol (≥99.9%), methanol (HPLC grade), formic acid (HPLC grade), and deionized water (HPLC grade).

### 2.2 Plant Material and Preliminary Preparation

Rye (*S. cereale L*.) plants representing six genetically distinct varieties were used in this study: Cl 98 (Hungary), Slapske (Hungary), Zidlochovicke Panis (Czech Republic), Ceske Normalni (Hungary), Bates (USA), and SU Stakkato (Germany, hybrid). Seeds of Cl 98, Slapske, Z. Panis, C. Normalni, and Bates were obtained from the National Small Grains Collection (NSGC, USDA), while SU Stakkato was provided by Saaten-Union (Hybro Saatzucht GmbH & Co. KG). Plants were cultivated under field conditions in Córdoba, Spain, during the 2022 growing season. After harvest, straw was separated from the grain, air-dried, and milled using a knife mill to pass through a 1 mm sieve. The ground material was stored in airtight containers at room temperature until further analysis.

### 2.3 Chemical Composition of Rye Straw

To determine the chemical composition, extractives were first removed to isolate the structural components of the cell wall. Approximately 40–50 g of ground straw was sequentially extracted in a Soxhlet apparatus with acetone, methanol, and water (8 h each). Extracts were concentrated using a rotary evaporator, and extractives contents were determined gravimetrically. The total lignin content of extractive-free straw was calculated as the sum of Klason lignin and acid-soluble lignin, corrected for protein and ash contents, following TAPPI UM 250 and T222 om-88 protocols.^23^ Ash content was measured gravimetrically after incineration at 600 °C for 6 h. Protein content was estimated from nitrogen content determined with a LECO CHNS-932 elemental analyzer, applying a conversion factor of 6.25.^24^ Holocellulose content (cellulose + hemicelluloses) was determined by the acid chlorite method.^25^ Hemicelluloses were subsequently removed by alkali extraction,^25^ and cellulose was calculated by difference. All analyses were performed in duplicate.

### 2.4 Isolation of Milled-Straw Lignin (MSL)

Extractive-free straw was further ground into fine powder (<1 μm) using a Retsch PM 100 planetary ball mill at 400 rpm for 5 h (effective milling time). Lignin was isolated according to the Björkman method,^22^ involving repeated extractions with dioxane–water (96:4 v/v). Combined extracts were concentrated under reduced pressure and subjected to repeated precipitation and resuspension cycles to yield purified milled straw lignin (MSL). Experimental details are provided in the Supporting Information. The MSL yield was typically 12–15% relative to the total lignin content of the original straw.

### 2.5 Pyrolysis–Gas Chromatography/Mass Spectrometry

Analytical pyrolysis of MSL (∼0.5 mg) was carried out at 500 °C using a Frontier 3030 microfurnace pyrolyzer (Fukushima, Japan) coupled to an Agilent 7820A gas chromatograph and 5975 mass spectrometer equipped with a DB-1701 fused-silica capillary column. Operating conditions followed the protocol reported elsewhere.^26^ Pyrolysis with tetramethylammonium hydroxide (TMAH) was performed by mixing 0.5 mg of lignin with 10 μL of 25% TMAH in methanol prior to analysis. Compounds identification was based on comparison with published spectra,^27^ while relative abundances were determined as previously described.^26,28^ Detailed parameters are provided in the Supporting Information.

### 2.6 2D-Nuclear Magnetic Resonance Analyses

Approximately 40 mg of MSL was dissolved in 0.5 mL of deuterated dimethyl sulfoxide (DMSO-*d*_6_) in an NMR tube. HSQC and HMBC spectra were acquired at 300 K on a Bruker Avance III 500 MHz spectrometer equipped with a 5 mm TCI cryoprobe. Spectral acquisition parameters and processing details are given in the Supporting Information. Signal assignments were made according to literature data.^10,12,29^ Quantification of interunit linkages and lignin structural units followed established methodologies,^10,26,29^ which are detailed in Supporting Information.

### 2.7 Derivatization Followed by Reductive Cleavage (DFRC)

Chemical degradation of lignin was conducted following the Derivatization Followed by Reductive Cleavage (DFRC) protocol,^30^ and its modified variant using propionylated reagents (so-called DFRC′).^31^ The released lignin-derived products were analyzed by GC/MS under conditions detailed in the Supporting Information. Relative molar abundances were calculated from the molecular weights of their acetylated or propionylated derivatives.

### 2.8 Quantitative ^31^P NMR Spectroscopy

Quantitative ^31^P NMR analyses were performed in duplicate according to the phosphitylation procedure previously described.^32^ Phosphitylated samples were analyzed on a Bruker Avance NEO 500 MHz spectrometer under acquisition conditions reported previously,^13^ detailed in the Supporting Information. Signal assignments followed established literature.^32–34^ Hydroxyl groups were quantified using *N*-hydroxy-5-norbornene-2,3-dicarboximide (NHND, 97% purity) as internal standard.

### 2.9 Molecular Weight Distribution

Molecular weight distribution was determined by gel permeation chromatography (GPC). Acetylated MSL samples were dissolved in tetrahydrofuran (THF) and analyzed using a Shimadzu Prominence-i LC-2030 3D system equipped with a PLgel 5 μm MIXED-D (7.5 × 300 mm) column. Calibration and detailed procedures are provided in the Supporting Information.

### 2.10 Deep Eutectic Solvent (DES) Treatment

Deep eutectic solvent was prepared by mixing choline chloride (ChCl, ≥98%) with lactic acid (LA, >80%) at a 1:10 molar ratio (ChCl/LA 1:10), following a previously described procedure.^21^ Briefly, the mixture was heated at 60–80 °C under stirring (250 rpm) for 1 h until an homogeneous and transparent liquid was obtained. The prepared DES was stored in a sealed container at room temperature. For each experiment, 0.75 g of dry, ground rye straw was mixed with 6.75 g of the selected DES (solid-to-liquid ratio 1:10, w/w) and heated at 120 °C for 1 h under constant stirring (600 rpm). After treatment, the mixtures were cooled to room temperature, and 15 mL of ethanol/water (1:1 v/v) was added to facilitate phase separation. The solid residue was separated by filtration, yielding a first filtrate (LF1), washed again with ethanol/water (1:2 v/v, 50 mL) to obtain a second filtrate (LF2), and dried at 40 °C for 24 h. Both filtrates (LF1 and LF2) were analyzed by HPLC for tricin quantification. Lignins dissolved in the DES phase (DES-L) were recovered by precipitation with cold distilled water (500 mL) under continuous stirring. Additional assays evaluated the effects of mild acidification of the DES with 0.2% (v/v) H_2_SO_4_ and ultrasound-assisted extraction (UAE, 1 h at maximum power) prior to DES treatment. The DES formulation showing the highest tricin release was selected for subsequent process optimization.

### 2.11 HPLC Analysis of Tricin Recovered after DES Treatment

Liquid fractions (LF1 and LF2) were analyzed using a Shimadzu Prominence-i LC-2030C 3D system equipped with a diode-array detector (350 nm) and a Shim-pack GWS C18 column (5 μm, 250 × 4.6 mm). The HPLC method was adapted from previous studies.^35,36^ The mobile phases were water (A) and methanol (B), both containing 0.1% formic acid. The gradient program was: 0–30 min, 40–90% B; 30–40 min, 90–40% B; followed by 5 min re-equilibration at 40% B. Flow rate was 0.8 mL/min, injection volume 10 μL, and column temperature 30 °C. Tricin was identified by retention time and UV spectra compared to authentic standards and quantified using a calibration curve (*y* = 75 870× −1 × 10^6^, *R* ^2^ = 0.9942). The total tricin content was calculated as the sum of the amounts detected in LF1 and LF2.

### 2.12 Calculation of Tricin Release and Lignin Recovery

Tricin release (mg/g) and lignin recovery (g/g) were calculated according to eqs 1 and ([Disp-formula eq2]), respectively

1 $$tricin\;release\;\left(mg/g\right)=\frac{mass\;of\;tricin\;in\;liquid\;fractions\;\left(mg\right)}{rye\;straw\;sample\;\left(g\right)}$$

2 $$lignin\;recovery\;\left(g/g\right)=\frac{mass\;of\;recovered\;lignin\;\left(g\right)}{mass\;of\;lignin\;in\;rye\;straw\;\left(g\right)}$$

## 3 Results and Discussion

### 3.1 Chemical Composition of the Different Rye Straw Varieties

The major chemical constituents of the rye straws were quantitatively determined, including extractives (acetone-, methanol-, and hot water-soluble fractions), total lignin (comprising Klason and acid-soluble lignins), holocellulose (cellulose and hemicelluloses), protein, and ash. As summarized in Table 1, the six rye varieties exhibited comparable compositional profiles. Holocellulose constituted the predominant fraction, accounting for 60.4–61.9% of the dry biomass. Within this fraction, cellulose was the major component (43.4–44.4%), while hemicelluloses contributed 16.0–17.9%. Lignin was the next most abundant constituent, contributing up to 14.2–15.4% of the dry biomass. Most of the lignin occurred as acid-insoluble lignin (Klason lignin) (12.3–13.6%), while acid-soluble lignin made up 1.6–1–9%. Methanol- and water-soluble extractives were present in significant amounts (6.3–8.3% and 6.4–7.3%, respectively), whereas acetone-soluble extractives were detected at lower levels (2.1–3.1%). Protein and ash contents were relatively low, accounting for 2.4–3.4% and 4.0–4.7% of the total dry mass, respectively. Overall, these compositional values are consistent with those reported for other rye varieties,^37^ as well as for other cereal straws, such as wheat and oat.^10,38^

**Table 1:** Percentage Abundance of the Main Straw Components in Rye Varieties^a^

	Cl 98	Slapske	Z. Panis	C. Normalni	Bates	SU Stakkato
**total extractives**	**17.1 ± 0.6**	**16.1 ± 0.8**	**17.3 ± 0.5**	**17.5 ± 1.0**	**16.3 ± 0.8**	**15.7 ± 0.7**
acetone	2.4 ± 0.3	2.3 ± 0.3	3.1 ± 0.1	2.4 ± 0.3	2.6 ± 0.3	2.1 ± 0.2
methanol	8.3 ± 0.1	6.9 ± 0.3	7.3 ± 0.2	7.9 ± 0.3	6.9 ± 0.4	6.3 ± 0.2
hot-water	6.4 ± 0.2	6.9 ± 0.2	6.9 ± 0.2	7.2 ± 0.4	6.8 ± 0.1	7.3 ± 0.3
**total lignin**	**14.7 ± 0.6**	**15.4 ± 0.3**	**15.2 ± 0.5**	**14.2 ± 0.6**	**14.9 ± 0.7**	**15.2 ± 0.3**
Klason lignin	13.0 ± 0.5	13.6 ± 0.2	13.5 ± 0.4	12.3 ± 0.6	13.3 ± 0.7	13.3 ± 0.3
acid-soluble lignin	1.7 ± 0.1	1.8 ± 0.1	1.7 ± 0.1	1.9 ± 0.0	1.6 ± 0.0	1.8 ± 0.0
**holocellulose**	**61.7 ± 2.2**	**61.4 ± 0.4**	**60.4 ± 3.3**	**61.3 ± 2.9**	**61.9 ± 0.6**	**61.5 ± 0.8**
hemicelluloses	17.9 ± 0.8	17.6 ± 0.1	16.0 ± 1.8	17.9 ± 2.2	17.7 ± 0.1	17.4 ± 0.7
α-cellulose	43.8 ± 1.4	43.8 ± 0.3	44.4 ± 1.5	43.4 ± 0.7	44.2 ± 0.5	44.1 ± 0.1
**proteins**	**2.5 ± 0.1**	**2.5 ± 0.0**	**2.7 ± 0.7**	**2.4 ± 0.4**	**2.4 ± 0.1**	**3.4 ± 0.7**
**ashes**	**4.0 ± 0.1**	**4.6 ± 0.1**	**4.4 ± 0.2**	**4.7 ± 0.0**	**4.5 ± 0.3**	**4.2 ± 0.1**

a Average of two replicates and expressed as percentage of dry weight.

Although the lignin content strongly influences biomass recalcitrance, its composition and structural features are equally important in determining biomass degradability. Therefore, a detailed understanding of the lignin structure is essential for accurately evaluating biomass resistance and optimizing its valorization in biorefinery processes. To investigate the lignin composition and structure in these rye straw varieties, native-like lignins were isolated from the different extractive-free rye straws using a neutral solvent-based method.^22^ The resulting milled straw lignins (MSL) are generally regarded as a close structural representative of native lignin within the plant cell wall.^39^ The isolated MSLs were subsequently characterized using advanced spectroscopic and analytical techniques to elucidate their chemical composition and structural features.

### 3.2 Analysis of Lignin Composition through Py-GC/MS

Rye MSLs were first analyzed using Py-GC/MS, a technique well-suited for characterizing lignin, as well as the occurrence of *p*-hydroxycinnamates (ferulates and *p*-coumarates). The resulting chromatograms are shown in Figure 1, while the identified phenolic compounds along with their relative molar abundances are detailed in Table 2.

**Figure 1:**
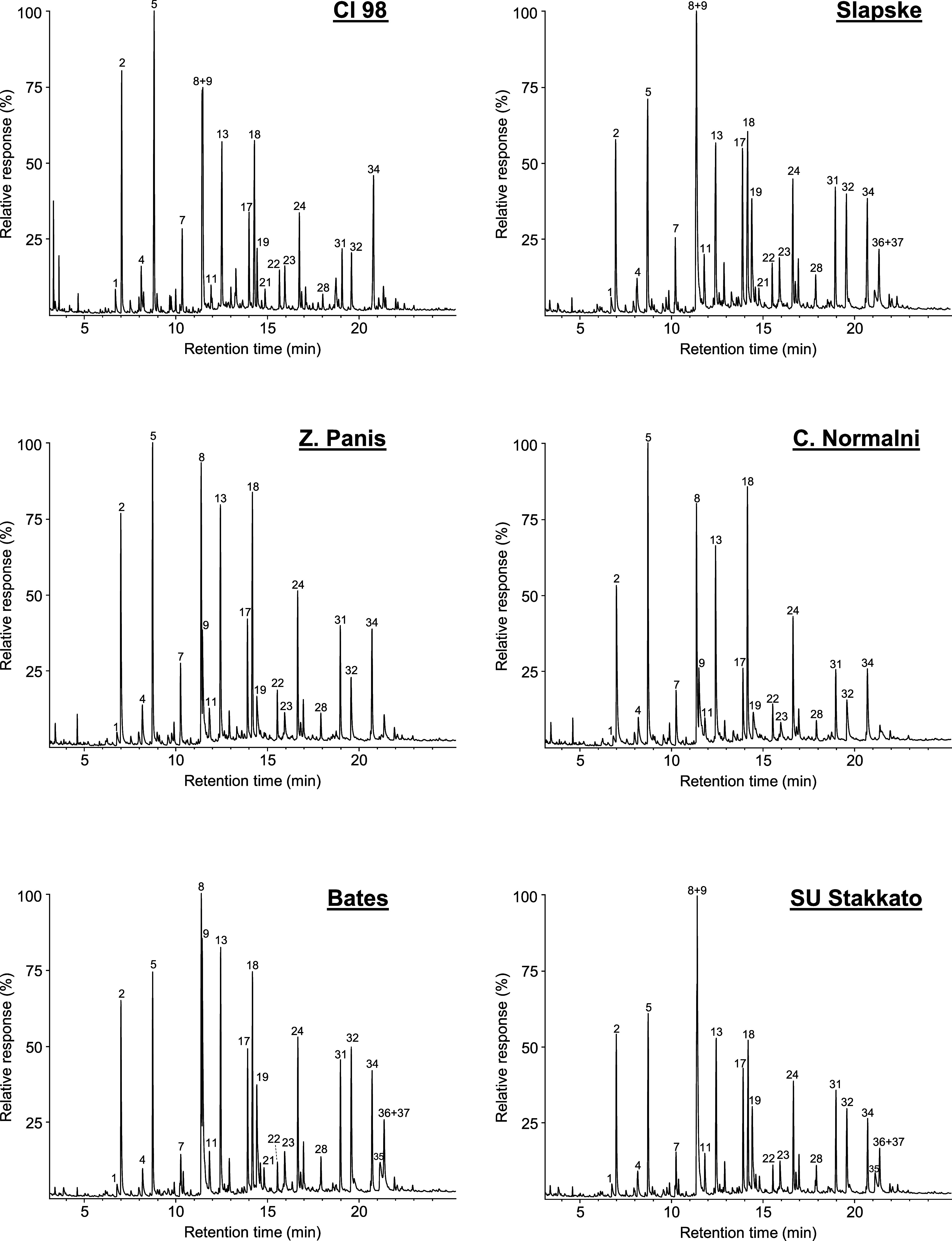
GC-MS pyrograms of the MSLs isolated from the different rye varieties. The identities and relative abundances of compounds released upon pyrolysis are listed in Table 2

**Table 2:** Identities and Relative Molar Abundances (%) of the Main Phenolic Compounds Identified Among the Pyrolysis Products of Rye MSLs^a^,^b^

N°	compound	origin^#^	Cl 98	Slapske	Z. Panis	C. Normalni	Bates	SU Stakkato
1	phenol	H	1.8	1.4	1.4	1.4	1.3	1.3
2	guaiacol	G	11.8	8.1	10.7	11.2	8.2	8.2
3	3-methylphenol	H	0.9	0.4	0.7	0.4	0.3	0.6
4	4-methylphenol	H	2.7	2.5	2.1	1.9	1.8	2.5
5	4-methylguaiacol	G	14.7	9.6	12.4	14.0	7.6	8.3
6	4-ethylphenol	H	1.0	0.3	0.5	0.6	0.3	0.5
7	4-ethylguaiacol	G	3.1	2.5	3.1	2.8	1.5	1.8
8	4-vinylphenol	H/*p*C	9.1	12.8	8.1	7.8	14.9	16.5
9	4-vinylguaiacol	G/FA	10.0	7.7	10.4	10.7	9.1	8.4
10	eugenol	G	0.7	1.2	0.8	0.7	0.9	1.0
11	4-propylguaiacol	G	0.3	0.7	0.6	0.7	0.4	1.0
12	4-allylphenol	H	0.1	0.3	0.5	0.4	0.2	0.4
13	syringol	S	8.3	6.9	9.2	9.3	8.3	7.0
14	*cis*-4-propenylphenol	H	0.1	0.2	0.2	0.2	0.1	0.2
15	*cis*-isoeugenol	G	0.7	1.2	0.8	0.7	0.9	0.9
16	*trans*-4-propenylphenol	H	0.3	0.5	0.7	0.6	0.4	0.6
17	*trans*-isoeugenol	G	3.4	5.2	3.8	3.1	4.0	4.5
18	4-methylsyringol	S	7.2	6.9	8.5	9.8	6.2	5.8
19	vanillin	G	2.3	4.8	2.6	2.3	5.2	4.5
20	propyne-guaiacol	G	0.4	0.5	0.3	0.2	0.8	0.7
21	propyne-guaiacol	G	0.4	0.4	0.3	0.3	0.6	0.7
22	4-ethylsyringol	S	1.2	1.1	1.4	1.2	0.7	0.9
23	acetovanillone	G	2.0	1.7	1.1	1.3	1.6	1.3
24	4-vinylsyringol	S	3.3	4.0	4.5	4.6	4.2	4.0
25	guaiacylacetone	G	0.6	0.8	0.8	0.7	0.7	0.8
26	4-allylsyringol	S	0.4	0.8	0.8	0.7	0.8	0.8
27	propiovanillone	G	0.2	0.3	0.3	0.3	0.1	0.2
28	*cis*-4-propenylsyringol	S	0.4	0.8	0.7	0.6	0.8	0.9
29	propyne-syringol	S	0.1	0.3	0.1	0.1	0.2	0.7
30	propyne-syringol	S	0.1	0.2	0.1	0.1	0.2	0.4
31	*trans*-4-propenylsyringol	S	1.7	3.3	3.2	2.5	3.3	3.3
32	syringaldehyde	S	2.1	4.1	2.8	2.9	4.8	3.7
33	*cis*-coniferyl alcohol	G	0.1	0.4	0.3	0.2	0.6	0.7
34	acetosyringone	S	6.1	3.6	3.7	3.5	3.3	2.7
35	*trans*-coniferyl alcohol	G	0.5	1.4	0.2	0.3	2.2	1.7
36	*trans*-coniferaldehyde	G	1.1	2.2	1.5	0.8	1.6	0.7
37	syringylacetone	S	0.3	0.2	0.1	0.6	1.6	1.3
38	propiosyringone	S	0.3	0.4	0.5	0.4	0.3	0.3
39	syringyl vinyl ketone	S	0.2	0.3	0.2	0.2	0.1	0.2
								
		H (%)*	8.8	7.4	8.0	7.1	6.0	8.7
		G (%)*	54.5	54.3	51.4	51.4	51.3	52.2
		S (%)*	36.7	38.3	40.6	41.5	42.7	39.1
		S/G ratio	0.67	0.71	0.79	0.81	0.83	0.75

a #Abbreviations: H, *p*-hydroxyphenyl units; G, guaiacyl units; S, syringyl units; *p*C, *p*-coumaric acid; FA, ferulic acid.

b *Calculated without considering 4-vinylphenol (8), 4-vinylguaiacol (9) and 4-vinylsyringol (24).

Pyrolysis of rye MSLs released a series of compounds derived from the three primary lignin monomers: *p*-hydroxyphenyl (H), guaiacyl (G), and syringyl (S). Among these, the most abundant were guaiacol (2), 4-methylguaiacol (5), 4-vinylguaiacol (9), 4-vinylphenol (8), syringol (13), 4-methylsyringol (18), acetosyringone (34), *trans*-isoeugenol (17), 4-vinylsyringol (24), and vanillin (19). In principle, the H:G:S lignin composition can be determined from the relative abundance of the phenolic compounds released during Py-GC/MS. However, the lignins from grasses, including cereal straws, are known to incorporate *p*-hydroxycinnamates such as *p*-coumaric acid (*p*C) and ferulic acid (FA).^9^ During pyrolysis, these compounds undergo decarboxylation forming 4-vinylphenol and 4-vinylguaiacol, respectively,^40,41^ which may be misinterpreted as products derived from genuine lignin components. Thus, accurate determination of the S/G ratios and the overall H:G:S lignin composition requires clarifying the origin of these pyrolysis products. This was achieved by using TMAH-assisted pyrolysis, in which in situ methylation of carboxylic groups effectively prevents decarboxylation during pyrolysis and releases the fully methylated products.^40,42^ The Py-TMAH-GC/MS chromatograms of the different rye straws (Figure S1) exhibited two prominent peaks corresponding to the fully methylated derivatives of *p*C and FA, characteristic markers of grass lignins.^10,13,26,43,44^ These results confirmed the presence of *p*-hydroxycinnamates in these lignins and indicated that a major fraction of the 4-vinylguaiacol and 4-vinylphenol released by conventional pyrolysis originates from them rather than from genuine lignin structural units. Consequently, it is obvious that these compounds cannot be used for the determination of the H:G:S compositional analysis. The H:G:S lignin composition was, therefore, estimated by ignoring the 4-vinylphenol and 4-vinylguaiacol (along the corresponding 4-vinylsyringol), and the results are reported in Table 2.

Py-GC/MS analysis revealed that rye MSLs were predominantly composed of G units, accounting for 51.3–54.5% of the total lignin-derived products, followed by S units (36.7–42.7%) and minor amounts of H units (6.0–8.8%), resulting in S/G ratios ranging from 0.67 to 0.83. This compositional pattern closely aligns with lignin profiles previously reported for other cereal straws such as oat and wheat straws.^10,38^ Among the rye varieties, the Cl 98 exhibited the lowest S/G ratio (0.67), whereas Bates variety showed the highest S/G ratio (0.83) (Table 2).

### 3.3 Structural Characteristic of Rye Straw Lignin Determined by 2D-NMR

The lignin monomeric composition and interunit linkages distribution of rye MSLs were investigated using 2D-HSQC-NMR spectroscopy. The HSQC spectra of the lignins from the different rye straw samples are shown in Figure 2, while the main lignin substructures and aromatic units identified are shown at the bottom. For clarity, the HSQC spectra were divided into two regions: the aliphatic-oxygenated regions (δ_C_/δ_H_ 48–90/2.5–5.8 ppm), and the aromatic/unsaturated regions (δ_C_/δ_H_ 90–150/5.3–8.0 ppm). The different lignin correlation signals observed in the HSQC spectra of the MSLs and their corresponding assignments are listed in Table S1.

**Figure 2:**
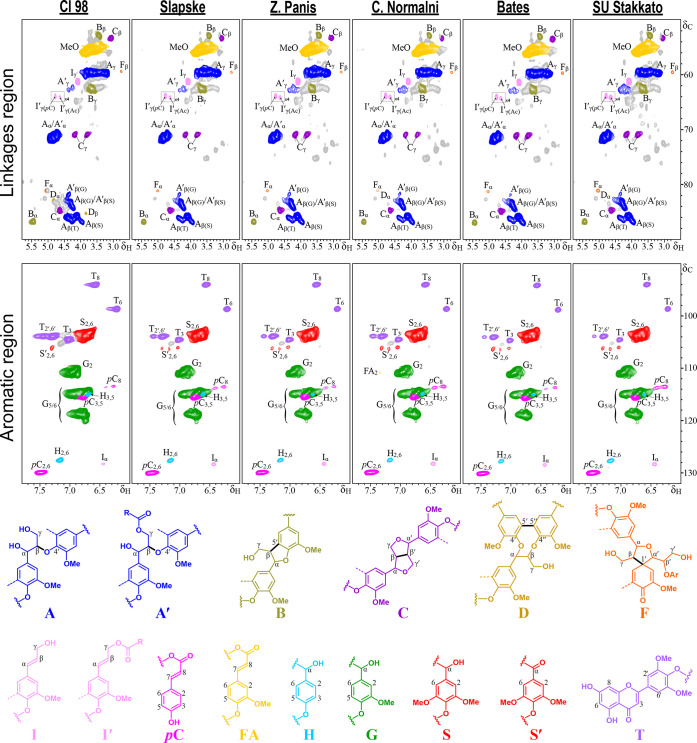
Aliphatic-oxygenated (δ_C_/δ_H_ 50–100/2.5–5.8, top) and aromatic/unsaturated (δ_C_/δ_H_ 90–132/6.0–7.8, bottom) regions from the HSQC spectra of the MSL isolated from selected rye straw samples. Lignin structures identified: β–*O*–4′ alkyl-aryl ethers (A), γ-acylated β–O–4′ alkyl-aryl ethers (A′), β–5′ phenylcoumarans (B), β–β′ resinols (C), 5–5′ dibenzodioxocins (D), β–1′ spirodienones (F), cinnamyl alcohol end-groups (I), γ-acylated cinnamyl alcohol end-groups (I′), cinnamaldehyde end-groups (J), *p*-coumarates (*p*C), ferulates (FA), *p*-hydroxyphenyl units (H), guaiacyl units (G), syringyl units (S), Cα-oxidized syringyl units (S′), and tricin (T). The colors of each structure and its corresponding HSQC signals match to facilitate spectrum interpretation.

The aromatic/unsaturated region of the HSQC spectra allowed the identification of the main lignin units (Figure 2). Prominent signals corresponding to guaiacyl (G) and syringyl (S) units were observed, together with minor signals from *p*-hydroxyphenyl (H) units. In addition, signals from tricin (T) and *p*-hydroxycinnamates, specifically *p*-coumarates (*p*C) and ferulates (FA), were detected, along with signals corresponding to cinnamyl alcohol (I) end-groups. Semiquantitative analysis of the HSQC spectra indicated that rye straw lignin comprised mostly G units (52.3–61.4% of all lignin units) followed by S units (35.2–45.2%), and lower levels of H unis (2.5–3.4%), resulting in an S/G ratio in the range of 0.57–0.86 (Table 3). Among the rye varieties, Cl 98 displayed the lowest S/G ratio (0.57), whereas Bates exhibited the highest value (0.86), in good agreement with the Py-GC/MS results. *p*C accounted for 5.4–7.9% of the total lignin units (H + G + S = 100), whereas FA contributed 1.5–2.7%. But more importantly, tricin content was remarkably high, particularly in the Cl 98 rye variety where it represented up to 21.1% of the total lignin units, followed by Slapske (11.6%), Zidlochovicke Panis (11.1%), Ceske Normalni (9.8%), Bates (8.1%), and SU Stakkato (7.5%).

**Table 3:** HSQC Semiquantitative Analysis of Rye MSLs, Including the Lignin Inter-Units Linkages, Terminal-Groups, γ-Acylation Degree, Structural Units, as Well as Ferulate, *p*-Coumarates and Tricin Moieties

	Cl 98	Slapske	Z. Panis	C. Normalni	Bates	SU Stakkato
linkages (%)^a^						
β–*O*–4′ alkyl-aryl ethers (A/A′)	78.4	78.2	78.2	78.7	79.1	78.8
β–5′ phenylcoumarans (B)	10.9	11.1	11.8	11.3	10.9	12.2
β–β′ resinols (C)	5.4	5.7	4.7	4.1	4.9	4.2
5–5′ dibenzodioxocins (D)	1.9	1.5	1.5	1.5	1.5	1.6
β–1′ spirodienones (F)	3.4	3.7	3.7	4.3	3.6	3.1
end-groups (%)^a^						
cinnamyl alcohol end-groups (I/I′)	5.0	7.3	8.7	8.6	7.4	8.7
γ-acylation degree (%)	5.0	7.5	7.4	7.4	7.1	9.9
aromatic units						
H (%)	3.4	3.1	2.9	2.5	2.5	2.6
G (%)	61.4	56.4	54.2	53.5	52.3	54.8
S (%)	35.2	40.5	42.9	44.0	45.2	42.6
S/G ratio	0.57	0.72	0.79	0.82	0.86	0.78
Tricin (T, %)^b^	21.1	11.6	11.1	9.8	8.1	7.5
*p*-Coumarates (*p*C, %)^b^	5.4	6.5	6.2	7.9	6.2	7.4
Ferulates (FA, %)^b^	1.5	1.9	1.7	2.7	1.8	2.0

a Estimated as a percentage of the total linkages (A-F).

b T and *p*C contents estimated as percentages of lignin content (H + G + S = 100).

The aliphatic-oxygenated region of the HSQC spectra provided detailed insights into the interunit linkages (Figure 2). The most prevalent linkages were the β–*O*–4′ alkyl-aryl ethers (A), accounting for 78.2–79.1% of all identified linkages, followed by β–5′ phenylcoumarans (B, 10.9–12.2%), and β–β′ resinols (C, 4.1–5.7%). Other linkages were present at lower levels, including 5–5′ dibenzodioxocins (D, 1.5–1.9%), and β–1′ spirodienones (F, 3.1–4.3%), while cinnamyl alcohol end-groups (I) were also detected (5.0–8.7%).

Notably, distinct correlation signals corresponding to β–*O*–4′ ether linkages involving tricin units (A_β(T)_) were clearly observed in the HSQC spectra, providing direct spectroscopic evidence for the incorporation of tricin into the lignin macromolecular structure. Furthermore, long-range 2D-Heteronuclear Multiple–Bond Correlation (HMBC) NMR analysis provided unequivocal evidence that tricin is covalently linked to the lignin backbone. A clear correlation between the C_4__′_ carbon of tricin and the H_β_ protons of the β–*O*–4′ ether linkage (Figure 3) confirms the presence of a 4′–*O*–β ether bond established between tricin and lignin units, as observed in other cereal straws.^10,13^

**Figure 3:**
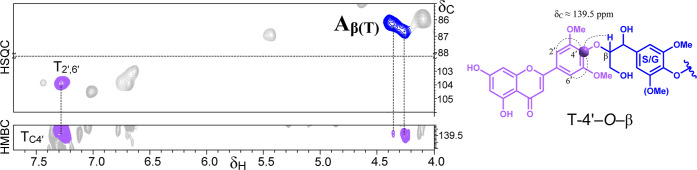
Selective regions of the HMBC spectrum of rye Cl 98 MSL. Correlations between the tricin C4′ signal (δ_C_ ∼139.5 ppm) and the β-protons of monolignol side-chains participating in β–*O*–4′ linkages. Corresponding HSQC regions displaying C_β_/H_β_ correlations of β–*O*–4′ alkyl–aryl ethers and the T_2′,6′_ correlations are also included.

The HSQC spectra also indicated that the γ–OH groups of the lignin side-chains were partially acylated, with an estimated acylation degree of 5–10%. The presence of significant amounts of *p*C, as evidenced by Py-TMAH and 2D-HSQC-NMR, suggests that *p*C could be the primary group acylating the γ–OH in these lignins, as reported for other cereal straws.^10,26,38^ This is further supported by the characteristic HSQC correlation signals assigned to *p*-hydroxycinnamyl alcohol end-groups acylated with *p*C (I_γ(*p*C)_) at δ_C_/δ_H_ 63.9/4.77 ppm. In addition, a distinct signal assigned to *p*-hydroxycinnamyl alcohol end-groups acylated with acetate groups (I_γ(Ac)_) was also clearly observed at δ_C_/δ_H_ 64.1/4.63 ppm, confirming that both *p*C and acetyl groups participate in γ–OH acylation position in these lignins.

### 3.4 Nature of the Groups that Acylate the γ–OH of the Lignin Side-Chains

To further confirm the identity of the γ-acylating groups, long-range 2D-HMBC NMR experiments were conducted. The HMBC spectra revealed two distinct carbonyl carbon signals (Figure 4). The signal at δ_C_ 166.0 ppm showed correlations with *p*C protons, whereas the signal at δ_C_ 169.5 ppm correlated with acetate methyl protons. Both carbonyl signals also showed correlations with protons in the δ_H_ 4.0–5.0 ppm range, confirming esterification at the γ-position of the lignin side chain. These correlations provided unequivocal evidence that pC and Ac are responsible for partial γ-acylation of lignin in rye straw.

**Figure 4:**
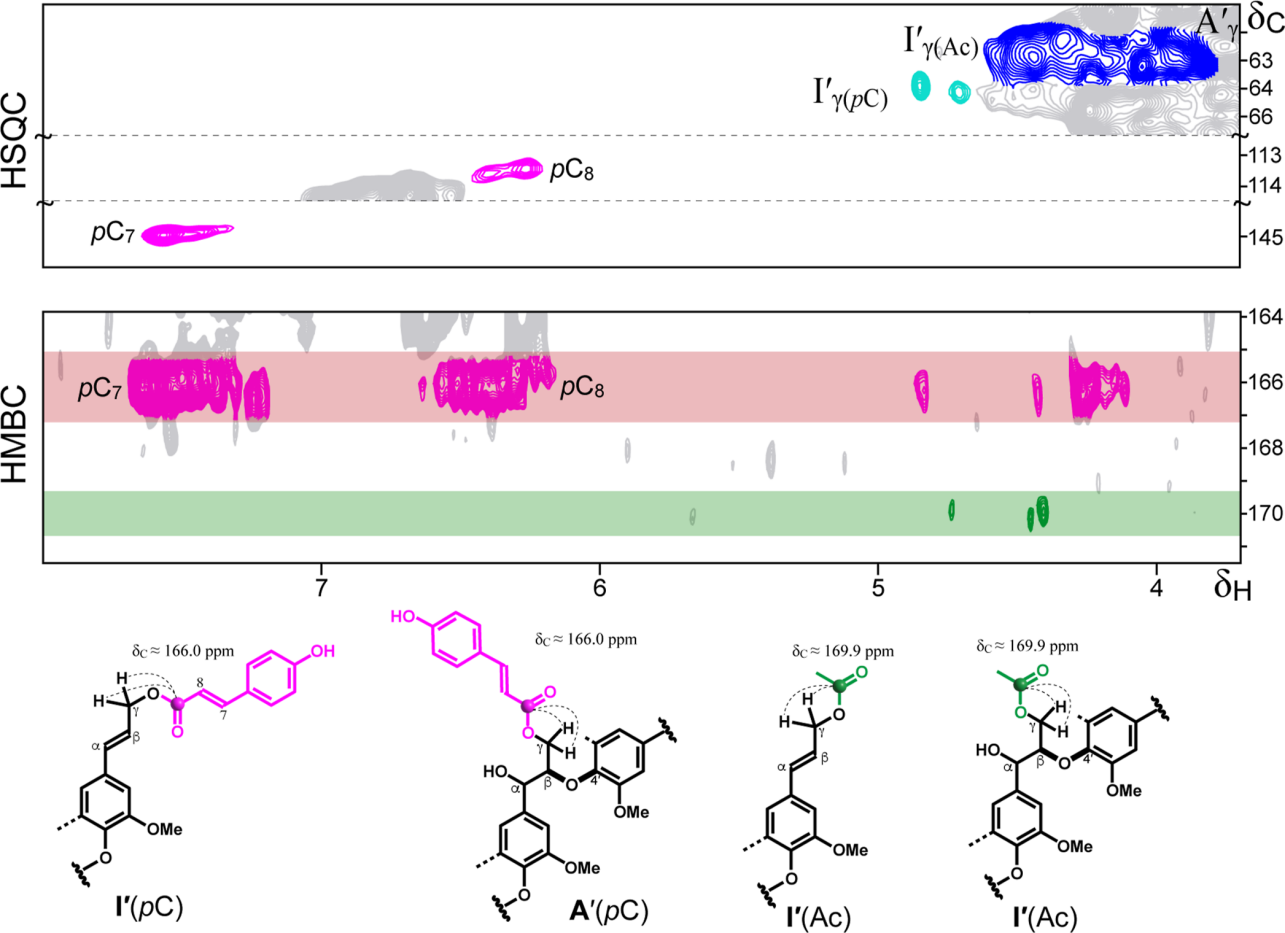
Selective regions of the HMBC spectrum of rye Cl 98 MSL. Main long-range correlations identifying carbonyl carbons of groups acylating the γ–OH of the lignin side-chains. Signals at δ_C_ ∼166.0 ppm correspond to *p*-coumarates (*p*C), whereas signals at δ_C_ ∼169.9 ppm indicate acetates (Ac). Relevant HSQC regions displaying the C_γ_/H_γ_ correlations of acylated lignin (δ_C_ 61–67 ppm) and the C_8_/H_8_ (δ_C_ 113–114 ppm) and C_7_/H_7_ (δ_C_ 144–145 ppm) signals of *p*C are also shown.

Further information on the nature and extent of γ–OH acylation was obtained using DFRC, a chemical degradation method that cleaves ether linkages, predominantly the β–*O*–4 bonds, the most abundant in lignin, while preserving ester bonds, including those involved in γ-acylation.^30,45^ DFRC selectively releases lignin monomers originally linked through β–*O*–4 ether bonds, either bearing free γ–OH groups or γ-acyl substituents, thus reflecting their native state within the lignin macromolecular structure.^30,31^ The chromatograms of DFRC products from rye MSLs are shown in Figure 5. The main compounds released were the *cis*- and *trans*-isomers of *p*-hydroxyphenyl (*c*H, *t*H), guaiacyl (*c*G, *t*G), and syringyl (*c*S, *t*S) monomers (detected as their acetylated derivatives), arising from β–*O*–4 linked units with nonacylated γ–OH, and showing a predominance of the G- and S-type monomers. In addition, detectable amounts of the *cis*- and *trans*-isomers of the sinapyl dihydro-*p*-coumarate (*c*S_*p*C_, *t*S_*p*C_) were released, together with minor amounts of the corresponding guaiacyl analogues (*c*G_*p*C_, *t*G_*p*C_). The release of these monolignol conjugates provides unequivocal evidence that *p*C is one of the groups acylating the lignin side chain. On the other hand, and more importantly, DFRC also released noticeable amounts of the flavone tricin (T), which was more abundant in the lignin from the Cl 98 variety, in good agreement with the 2D-HSQC-NMR results.

**Figure 5:**
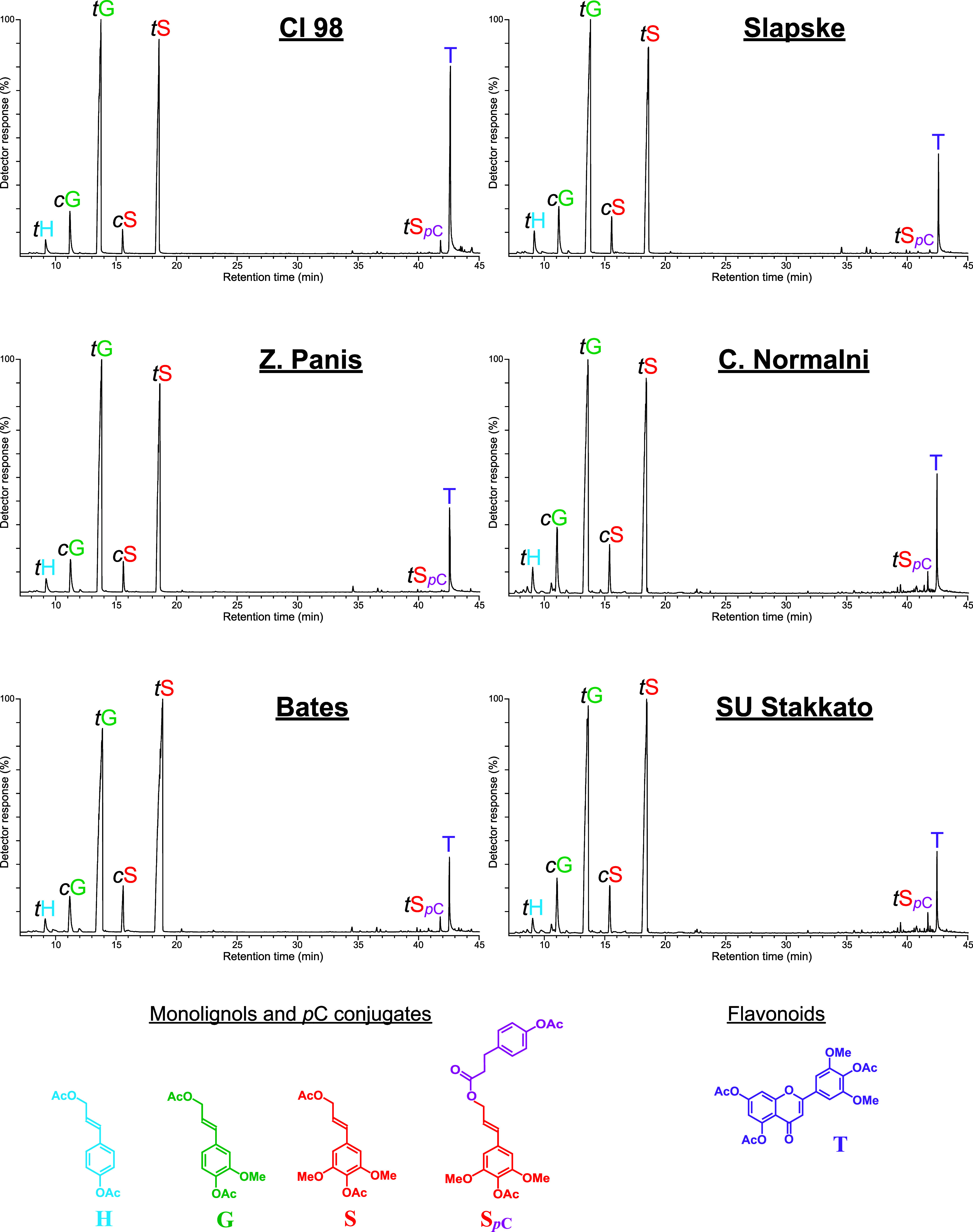
Reconstructed ion chromatograms (sum of the ions at *m*/*z* 192 + 222 + 252 + 400 + 330, which are characteristic of compounds H, G, S, S_*p*C_, and T, respectively) of DFRC degradation products released from the different rye straw lignins selected for this study. The structures of the identified compounds are represented at the bottom.

Acetate groups are also known to acylate the γ–OH of lignins in many plant species,^46^ and the presence of the characteristic correlations observed in the HSQC (signal at δ_C_/δ_H_ 64.1/4.63 ppm) and HMBC spectra above indicates that γ–OH acetylation also occurs in these lignins. However, the standard DFRC degradation protocol employs acetylating reagents and therefore cannot be used to assess the presence of native γ-acetylation. To overcome this limitation, an analogous protocol using propionylating reagents (DFRC′) was applied, allowing the specific detection of native acetylated units in the lignins.^31^ DFRC′ of rye MSLs released *c*H, *t*H, *c*G, *t*G, *c*S, and *t*S monomers (as their propionylated derivatives) originating from both acetylated and nonacetylated precursors (Figure S2). The chromatogram revealed low but unambiguous levels of native γ-acetylated units, mainly guaiacyl acetates (Gac), confirming the presence of native γ-acetylation in rye lignin, though in lower abundance than reported in other grasses.^38,44^

Quantitative analysis of DFRC and DFRC′ products (Table 4) revealed that G-units were predominant (47.2–61.4%), followed by S-units (35.8–51.0%), while H-units represented only a minor proportion (1.7–3.7%). These obtained data indicated that rye straw lignins were γ-acylated by both *p*C and Ac, in agreement with the NMR data. These results confirmed that *p*-coumaroylation in rye straw lignin is highly selective for S units, while acetates are mainly associated with G units, similarly to other cereals such as wheat, oats, and rice.^10,26,38^

**Table 4:** Relative Molar Abundance of H-, G-, and S-Lignin Units with Non-Acylated and Acylated γ–OH (with Ac and *p*-Coumarates) and Tricin (T), Estimated From DFRC (and DFRC′**)** Degradation of Rye MSLs


	H	G	G_ac_	G_*p*C_	S	S_ac_	S_*p*C_	T^a^	% G_ac_ ^b^	% G_*p*C_ ^c^	% S_ac_ ^d^	% S_*p*C_ ^e^
Cl 98	2.7	59.4	2.0	tr	35.1	0.2	0.5	12.5	3.3	tr	0.5	1.4
Slapske	3.3	58.2	2.0	tr	36.1	0.2	0.2	3.7	3.3	tr	0.4	0.4
Z. Panis	2.6	57. 1	2.3	tr	37.6	0.2	0.1	2.9	3.9	tr	0.6	0.3
C. Normalni	3.7	55.7	2.5	tr	37.4	0.3	0.5	3.7	4.4	tr	0.7	1.3
Bates	1.7	45.5	1.7	tr	50.5	0.2	0.3	2.2	3.7	tr	0.5	0.7
SU Stakkato	2.2	52.0	2.8	tr	42.4	0.2	0.4	2.5	5.1	tr	0.5	1.0

a T molar content referred as to the percentage of total lignin units (H + G + G_ac_ + G_*p*C_ + S + S_ac_ + S_*p*C_ = 100).

b % G_ac_ is the percentage of acetylated G units (G_ac_) with respect to the total G units (G + G_ac_ + G_*p*C_).

c % G_*p*C_ is the percentage of *p*-coumaroylated G units (G_*p*C_) with respect to the total G units (G + G_ac_ + G_*p*C_).

d % S_ac_ is the percentage of acetylated S units (S_ac_) with respect to the total S units (S + S_ac_ + S_*p*C_).

e % S_*p*C_ is the percentage of *p*-coumaroylated S units (S_*p*C_) with respect to the total S units (S + S_ac_ + S_*p*C_).

Finally, and more importantly, tricin was clearly released from all lignins and accounted for around 2.2–12.5% of the total lignin units, being more abundant in the variety Cl 98, as also advanced by the HSQC-NMR spectra (Figure 2).

### 3.5 ^31^P NMR Analysis of Rye Straw Lignin

To further elucidate the composition of rye straw lignins, the samples were analyzed by ^31^P NMR spectroscopy, a well-established technique for characterizing and quantifying hydroxyl functionalities in lignin, including carboxylic, aliphatic, and phenolic groups.^32^ This technique has also recently been shown to enable the detection and quantification of flavonoids such as tricin in different lignins.^33,34^ The ^31^P NMR spectra of the MSLs isolated from the different rye varieties, along with the assignment of the major resonances, are shown in Figure 6, whereas the quantitation of the various hydroxyl groups, including those of tricin, are presented in Table 5. The prominent signals within the range of 145.0–150.0 ppm corresponded to aliphatic hydroxyl groups in the lignin side chain, with values ranging from 3.69 mmol/g in Cl 98 variety to 4.97 mmol/g in Bates. Phenolic hydroxyl signals were observed in the 137.0–145.0 ppm region, arising from H-, G-, and S-lignin units, as well as from *p*C and tricin. Among these, G-type phenolic hydroxyls were the most abundant in all MSLs analyzed (0.57–0.70 mmol/g), whereas S-type hydroxyls were present in minor amounts (0.12–0.28 mmol/g). Carboxylic hydroxyl groups were detected at 133.5–134.5 ppm, but at comparatively lower levels (0.11–0.17 mmol/g). The signals from H-units and *p*C overlapped with that of tricin 5-OH in the 136–137 ppm region,^32^ preventing their individual quantification. In contrast, the tricin 7-OH resonance was well resolved and was therefore used for tricin quantification, which accounted for 0.24–0.54 mmol/g. Assuming that a stoichiometry between tricin and its 7-OH group is 1:1, that the MSLs are representative of the native lignin in rye straw, and considering the total lignin content (Klason + acid-soluble) of the dry biomass, it was possible to estimate the amount of tricin in the original rye straw based on the ^31^P NMR data.^13^ The ^31^P NMR analysis revealed that the rye straw samples contained considerable amounts of lignin-bound tricin (12.0–26.2 g/kg straw), with the Cl 98 variety exhibiting the highest content (Table 5).

**Figure 6:**
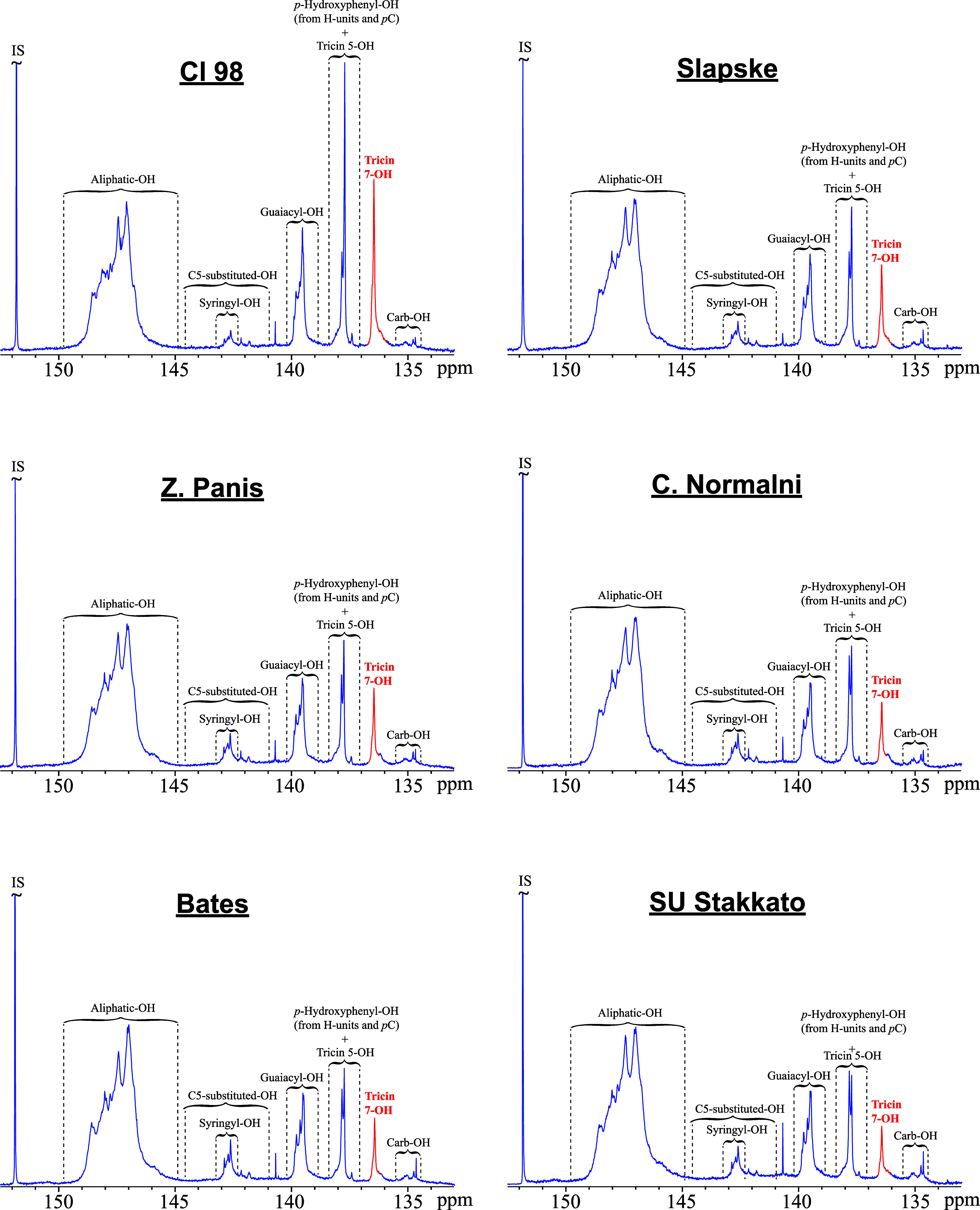
^31^P NMR spectra of rye MSL previously phosphitylated with TMDP and using NHND as an internal standard (IS) for quantification.

**Table 5:** Aliphatic, Phenolic (Syringyl + Guaiacyl + *p*-Hydroxyphenyl + C_5_-Substituted–OH + Tricin), and Carboxylic Hydroxyl Group Contents (mmol OH/G Lignin) in the MSLs Isolated from Rye, as Well the Tricin Content (g) Per kg of Dry Straw, as Determined by Quantitative ^31^P NMR

	content (mmol OH/g MWL)							
Variety	Aliph–OH	G–OH	S–OH	*p*-Ar–OH (H–OH + *p*C–OH)	C5-sub	Carb–OH	Tricin–OH	Tricin content
Cl 98	3.69	0.69	0.12	0.12	0.05	0.11	0.54	26.2
Slapske	4.14	0.62	0.19	0.17	0.07	0.16	0.31	15.8
Z. Panis	4.04	0.60	0.21	0.15	0.08	0.15	0.30	15.0
C. Normalni	4.45	0.60	0.23	0.21	0.09	0.15	0.29	13.6
Bates	4.97	0.70	0.28	0.22	0.09	0.17	0.27	13.3
SU Stakkato	4.21	0.57	0.21	0.17	0.08	0.16	0.24	12.0

A particularly notable observation was the absence of signals in the 142.0–143.0 ppm range, characteristic of the 4′–OH of free tricin,^33^ providing strong evidence that tricin is not present in free form in rye straw lignin. Instead, tricin is exclusively incorporated into the lignin polymer, in agreement with the 2D-HMBC data, which demonstrated that tricin is covalently integrated into the rye straw lignin through 4′–*O*–β ether linkages.

### 3.6 GPC Analyses of Rye Lignin

The weight-average (M_*w*_) and number-average (M_*n*_) molecular weights of the rye MSLs were determined by gel permeation chromatography (Table 6). The M_*w*_ values ranged from 4030 to 5410 g·mol^–1^, and the M_*n*_ values from 2200 to 2780 g·mol^–1^, resulting in low polydispersity indices (1.57–2.33) that indicate a high degree of molecular homogeneity. Similar M_*w*_ values have been reported for other tricin-rich lignins, including those from tritordeum straw and vanilla aerial roots.^13,34^

**Table 6:** Weight-Average (M_*w*_) and Number-Average (M_*n*_) Molecular Weights (g·mol^–1^), and Polydispersity (M_*w*_/M_*n*_) of Rye MSLs

	Cl 98	Slapske	Z. Panis	C. Normalni	Bates	SU Stakkato
M_*w*_	4030	4830	5130	5270	5410	5350
M_*n*_	2570	2620	2200	2590	2770	2780
polydispersity	1.57	1.84	2.33	2.03	1.95	1.92

Importantly, the GPC chromatograms showed no detectable peaks corresponding to free tricin or other low-molecular-weight compounds, confirming that tricin is not present in its free form but instead is covalently bound to the lignin macromolecular structure. This observation is consistent with NMR results and further support the role of tricin as a nucleation site for lignin polymerization. Since tricin can be incorporated into the lignin structure only through 4′–*O*–β coupling, it acts as a terminal unit that initiates a new lignin chain.^47^ Consequently, an increased tricin content promotes a high number of initiation events during polymer formation, leading to a larger population of shorter chains rather than favoring the elongation of existing ones. This explains why the Cl 98 variety, which is enriched in tricin, exhibits a lower M_w_ (4030 g·mol^–1^) and a reduced degree of polymerization.

### 3.7 Recovery of Lignin-Bound Tricin Using Deep Eutectic Solvents (DES)

The relative high content of lignin-bound tricin in Cl 98 rye straw prompted further investigation into environmentally benign extraction approaches using the DES ChCl/LA (1:10) for its recovery. This DES has been widely investigated for lignin extraction from lignocellulosic biomass due to its demonstrated efficiency in lignin solubilization and its ability to promote β–*O*–4 ether bond cleavage.^21,48–52^ The effectiveness of ChCl/LA arises from its dual functionality: choline chloride acts as a hydrogen bond acceptor, while lactic acid serves as both a proton donor and mild acid catalyst, together generating a dense hydrogen-bonding network and localized proton activity that facilitate ether bond cleavage, lignin depolymerization, and enhanced lignin solubilization.^48,53^ In addition, the chloride anion plays a key role in the depolymerization mechanism by acting as a nucleophile that accelerates β–*O*–4 bond cleavage.^21^ DES are particularly appealing due to their green connotation, but also because they enable effective lignin removal, fostering biomass valorisation for other applications (e.g., improved digestibility for biofuels production).^20^

To further increase tricin release, the DES system was supplemented with a small amount of sulfuric acid (0.2%), and ultrasound assisted extraction was also evaluated. A schematic overview of the process is shown in Figure S3, while the tricin yields in the soluble fraction and the corresponding lignin recovery for each treatment are summarized in Table 7.

**Table 7:** Results of Different DES Treatments on Tricin Release (mg/g Dry Biomass) and Lignin Recovery (g/g Dry Biomass) from Milled Rye Straw Samples (Cl 98)^a^

assay	DES (molar ratio)	Tricin released	lignin recovery
1	ChCl/LA (1:10)	1.92	0.40
2	ChCl/LA (1:10) + 0.2% H_2_SO_4_	1.69	0.41
3	ChCl/LA (1:10) + UAE	1.84	0.46
4	ChCl/LA (1:10) twice	2.34	0.26

a Experimental Conditions: Solid-to-Liquid Ratio 1:10, 120 °C, and 60 min.

Under the conditions tested, the ChCl/LA (1:10) system exhibited the highest tricin release (1.92 mg/g of straw) and lignin recovery of 0.40 g/g dry biomass, confirming its strong ability to disrupt β–*O*–4 linkages and efficiently release tricin moieties. Increasing the acidity by H_2_SO_4_ addition resulted in a lower tricin yield (1.69 mg/g) while providing a comparable lignin recovery (0.41 g/g). In contrast, ultrasound-assisted extraction (UAE) slightly improved lignin recovery (0.46 g/g) but did not enhance tricin release (1.84 mg/g). The results highlight ChCl/LA (1:10) as an effective and operationally straightforward DES for the selective release of tricin from Cl 98 rye straw lignin.

To further elucidate the structural alterations occurring in lignin during the treatment with ChCl/LA (1:10), the DES-derived lignin (DES-L) obtained from extractives-free straw was analyzed by 2D HSQC NMR spectroscopy. Additionally, a portion of this recovered lignin was subjected to a second with ChCl/LA (1:10) DES treatment to evaluate further tricin release. The lignin-rich fraction obtained after precipitation and filtration (DES-LR) was also characterized by 2D HSQC NMR, while the corresponding liquid fractions were analyzed by HPLC. The 2D HSQC NMR spectra of the lignin-enriched fractions (DES-L and DES-LR) provided clear evidence of tricin release and allowed monitoring of its progressive liberation during the DES treatments. A gradual reduction in the cross-peaks associated with lignin-bound tricin was observed as treatment proceeded, accompanied by an increase in the correlation signal corresponding to nonetherified (free) tricin (Tf_3_), which appears at a distinct chemical shift relative to the bound form (T_3_) (Figure 7). After the second ChCl/LA (1:10) DES treatment, the HSQC spectrum clearly showed that most tricin was present in its free form rather than as lignin-etherified tricin (Figure 7C), indicating extensive cleavage of tricin–lignin linkages and confirming the high efficiency of the DES-mediated extraction process. Consistent with these spectral data, HPLC analysis of the liquid fraction collected after the second DES treatment identified an additional 0.42 mg/g of free tricin, increasing the total tricin released to 2.34 mg/g rye straw (Table 7).

**Figure 7:**
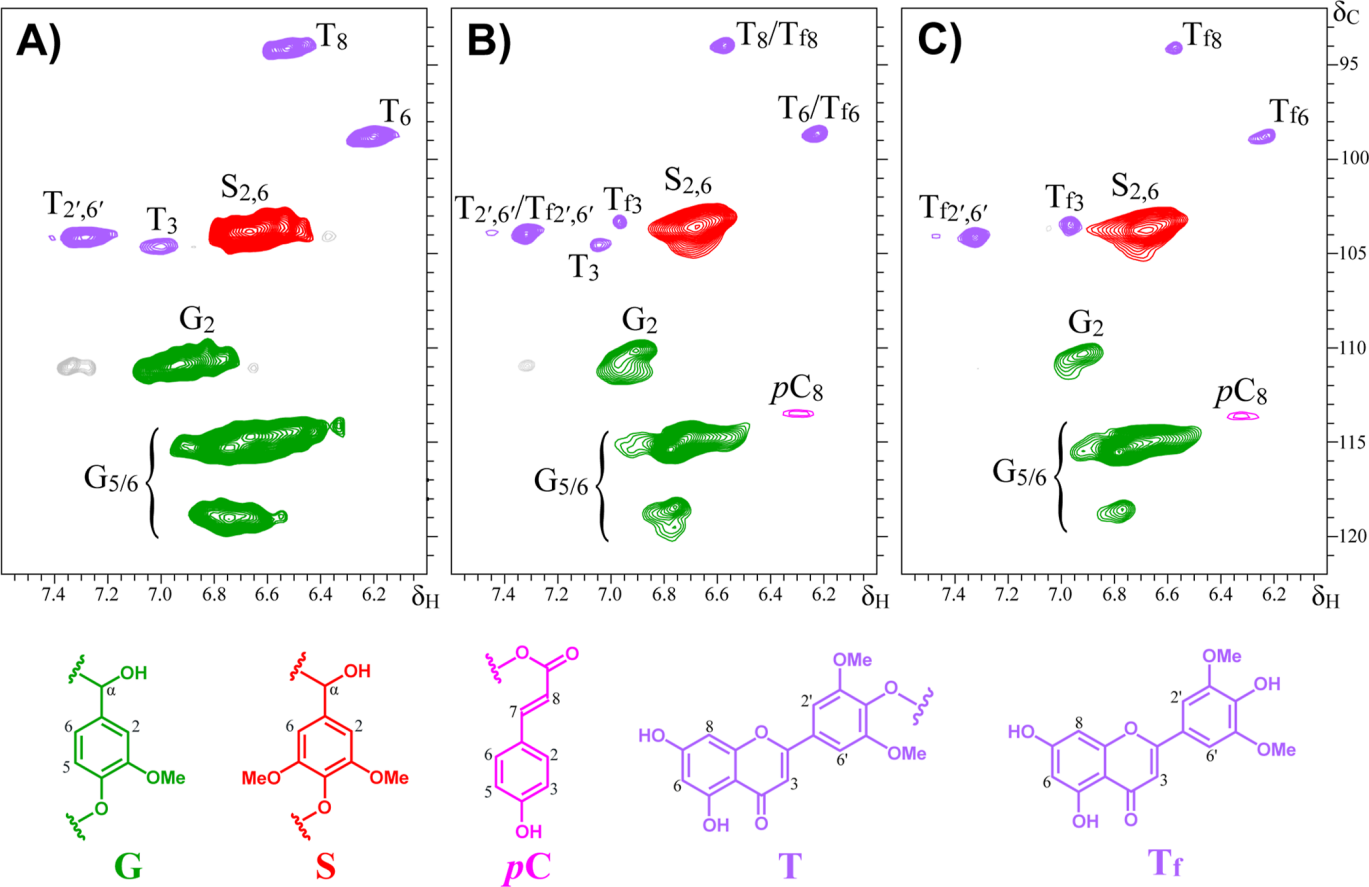
HSQC spectra of the initial Cl 98 material (A) and the lignin-enriched fractions DES-L (B) and DES-LR­(C).

The results underscore the potential of ChCl/LA based DES as selective and sustainable media for lignin depolymerization and the recovery of structurally intact, lignin-derived flavonoids such as tricin. 2D HSQC NMR analyses confirmed the progressive liberation of tricin from lignin during DES treatments, with most tricin present in its free form after a second pretreatment, demonstrating the high efficiency of the DES-mediated extraction process.

## 4 Conclusions

The lignin content and structural composition of the straw from different rye varieties were comprehensively characterized. The lignins (accounting for ∼ 14.2–15.4% of the straw) were slightly enriched in G-lignin units, with S/G ratios ranging from 0.57 to 0.86. Their structure was dominated by β–*O*–4′ alkyl-aryl ether linkages, which accounted for 78.2–79.1% of all identified interunit linkages, together with significant amounts of condensed linkages, including β–5′ phenylcoumarans (10.9–12.2%), β–β′ resinols (4.1–5.7%), 5–5′ dibenzodioxocins (1.5–1.9%), and β–1′ spirodienones (3.1–4.3%). But more importantly, rye straw lignins were found to be remarkably enriched in the flavone tricin, a compound of significant biological and industrial interest, with considerably variations in tricin content among cultivars. Among the varieties examined, Cl 98 exhibited the highest level of lignin-incorporated tricin. Given that tricin occurs as a terminal unit in the lignin polymer and is exclusively linked through relatively labile ether bonds, rye straw represents a promising renewable source of this high-value phenolic compound. Importantly, this study demonstrates for the first time that the deep eutectic solvent ChCl/LA (1:10) can efficiently cleave lignin ether linkages and release structurally intact tricin under mild, environmentally benign conditions. Together, these findings highlight the dual value of rye straw as a source of structurally distinct lignins and a promising platform for sustainable lignin valorization, underscoring the potential of DES-based extraction as an efficient and selective approach for the release of bioactive aromatic molecules, as tricin, from agricultural residues. There is still plenty of room to explore distinct biomass sources and DES to further improve the yields of these value-added compounds, integrated with the valorization of other biomass components.

## Supplementary Material



## Notes

Detailed information on the methodology used. Assignments of the ^13^C/^1^H correlation signals in the 2D HSQC spectra from rye MSL (Table S1) Py-TMAH chromatograms of the MSLs isolated from different rye varieties (Figure S1). GC/MS chromatograms of the DFRC′ degradation products of the MSLs (Figure S2). Flowchart illustrating the sequential steps of the deep eutectic solvent (DES) treatment (Figure S3). (PDF)
