# Supplementary material for: Rye Straw Lignin as a Promising Source of Tricin: Varietal Differences and Its Release Using Deep Eutectic Solvents
Source: ACS Sustain Chem Eng. 2026 Apr 10;14(16):7947–62. doi: 10.1021/acssuschemeng.6c02660 (PMC13127124; doi:10.1021/acssuschemeng.6c02660)
Supplement: Supplementary file 1 [file sc6c02660_si_001.pdf]

Supporting Information

Rye straw lignin as a promising source of tricin: Varietal differences and its release using deep eutectic solvents

Javier Benito[1], Raquel Cañadas[1], Francisco Barro[2], Ana Gutiérrez[1], André M. da Costa Lopes[3], Nalin Seixas[3], Sonia A.O. Santos[3], Armando J.D. Silvestre[3], José C. del Río[1], Jorge Rencoret[1*]

[1]Instituto de Recursos Naturales y Agrobiología de Sevilla, CSIC, Reina Mercedes 10, 41012 Seville, Spain

[2]Instituto de Agricultura Sostenible (IAS), CSIC, Av. Menéndez Pidal, S/N, 14004 Córdoba, Spain

[3]CICECO - Aveiro Institute of Materials and Department of Chemistry, University of Aveiro, Campus de Santiago, Aveiro 3810-193, Portugal

Summary:

- Detailed information on the methodology used (pp. S2–S4)
- Table S1. Assignments of the $^{13}C/^{1}H$ correlation signals in the 2D HSQC spectra from rye MSL (p. S5).
- Figure S1. Py-TMAH chromatograms of the MSLs isolated from different rye varieties (p. S6).
- Figure S2. GC/MS chromatograms of the DFRC′ degradation products of the MSLs (p. S7).
- Figure S3. Flowchart illustrating the sequential steps of the deep eutectic solvent (DES) treatment used for processing rye straw samples in this study (p. S8).
- References (p. S9)

Isolation of milled-straw lignin (MSL) from rye straw

The MSL preparation was obtained according to the classical procedure.[1] Extractive-free rye straw (around 70 g) was finely milled in a Retsch PM100 planetary ball mill (Restch, Haan, Germany) for 5 h at 400 rpm using a 500 mL agate jar and agate ball bearings (20 x 20 mm). The ball-milled samples were then extracted (3 × 24 h) with dioxane-water (96:4, v/v) (20 mL of solvent per gram of milled sample). The solution was centrifuged and the supernatant evaporated at 40 °C at reduced pressure. The residue obtained (raw MSL) was redissolved in a solution of acetic acid/water 9:1 (v/v) (20 mL solvent/g raw MSL). The solution was precipitated into water, and the residue was separated by centrifugation, milled in an agate mortar, and dissolved in a solution of 1,2-dichloromethane/ethanol (2:1, v/v). The mixture was then centrifuged to eliminate the insoluble material. The resulting supernatant was precipitated in diethyl ether, and the obtained residue was separated by centrifugation. This residue was then resuspended in petroleum ether and centrifuged again to obtain the final purified MSL fraction, which was dried under a current of N_2.

Pyrolysis coupled to gas chromatography and mass spectrometry (Py-GC/MS)

The pyrolysis of rye MSL (ca. 0.5 mg) was performed at 500 ºC in an EGA/PY-3030D microfurnace pyrolyzer (Frontier Laboratories Ltd., Fukushima, Japan) connected to a GC 7820A (Agilent Technologies, Inc., Santa Clara, CA) equipped with a DB-1701 fused-silica capillary column (30 m x 0.25 mm i.d., 0.25 µm film thickness) and an Agilent 5975 mass-selective detector (EI at 70 EV). The oven temperature was programmed from 50 ºC to 100 ºC at 20 ºC min^{-1} and then ramped to 280 ºC at a heating rate of 6 ºC min^{-1} and held for 5 min. The carrier gas was helium at 1 mL min^{-1}. For the pyrolysis in the presence of tetramethylammonium hydroxide (TMAH), around 0.5 mg of sample were mixed with 10 µL of TMAH (25% w/w, in methanol), and the pyrolysis was carried out as described above. The released compounds were identified by comparison of their mass spectra with those reported in the literature,[2] and when possible, by comparison with the retention times and mass spectra of our own collection of authentic standards. Molar peak areas were calculated for the released pyrolysis products, the summed areas were normalized, and the data for two repetitive analyses were averaged and expressed as percentages. The relative standard deviation for the pyrolysis data was below 10%. No attempt was made to calculate the response factor for every single compound released. However, for most of the lignin-derived phenols, the response factors are quite similar,[3] with the exception of vanillin, but this is a minor peak here.

Derivatization Followed by Reductive Cleavage (DFRC)

The DFRC was performed according to the originally developed method,[4–6] and the detailed explanation of the experimental procedure can be found elsewhere.[7] Rye MSL (5 mg) were stirred for two hours at 50 °C with acetyl bromide in acetic acid (8:92). The solvents and excess acetyl bromide were removed by rotary evaporation at reduced pressure. The products were then dissolved in dioxane/acetic acid/ water (5:4:1, v/v/v), and 50 mg of powdered Zn was added. After 40 min of stirring at room temperature, the mixture was transferred into a separatory funnel with dichloromethane and saturated ammonium chloride. The pH of the aqueous phase was adjusted to less than 3 by adding 3% HCl, the mixture vigorously mixed and the organic layer separated. The water phase

was extracted twice more with dichloromethane. The combined dichloromethane fractions were dried over anhydrous Na_2SO_4, and the filtrate was evaporated on a rotary evaporator. The residue was acetylated with 0.2 mL of acetic anhydride and 0.2 mL of pyridine for 1 h. In order to assess the presence of naturally acetylated lignin units, the DFRC method was slightly modified to use propionylating reagents instead of acetylating ones (so-called DFRC′), as previously described.[8,9] The lignin degradation products released by DFRC and DFRC′ were analyzed in a GCMS QP2020 instrument (Shimadzu Co., Kyoto, Japan) using a capillary column (DB5HT 30 m × 0.25 mm I.D., 0.1 µm film thickness). The oven temperature was heated from 140 (1 min) to 250°C at a rate of 3°C min^{-1}, then ramped at 10°C min^{-1} to 300°C, and maintaining the final temperature for 18 min. The injector temperature was set at 250°C while the transfer line was kept at 310°C. The carrier gas was helium (1 mL min^{-1} flow rate). The relative molar abundances of the released lignin degradation products

were determined using the molecular weights of their respective acetylated or propionylated compounds.

2D-NMR Analyses (detailed experimental conditions)

Rye MSL sample (~40 mg) were transferred into a 5-mm NMR tube and dissolved in 0.5 mL of deuterated dimethyl sulfoxide (DMSO-d_6). NMR experiments were carried out on a Bruker Avance III 500 MHz (Bruker, Karlsruhe, Germany) spectrometer fitted with a 5 mm TCI (triple cryoprobe inverse). These NMR analyses were conducted at the NMR facilities of the General Research Services of the University of Seville (SGI-CITIUS). Two dimension-Heteronuclear Single Quantum Coherence (2D-HSQC) NMR spectra were acquired at 300 K using an adiabatic pulse sequence (hsqcetgpsisp.2), which enabled a semiquantitative analysis of the different ^{13}C-^{1}H correlation signals. Spectra were acquired from 10 to 0 ppm in F2 (^{1}H) using 1676 data points for an acquisition time (AQ) of 145 ms, an interscan delay (D1) of 1 s, and from 165 to 0 ppm in F1 (^{13}C) using 256 increments of 32 scan, for a total experimental time of 2 h 40 min. The $^{1}J_{CH}$ coupling constant used was 145 Hz. HSQC spectra were processed using the Bruker TopSpin 4.2 software, and the following parameters: Gaussian apodization in ^{1}H (LB=-0.1 and GB=0.001) and a squared cosine bell in ^{13}C (LB=0.3 and GB=0.1). The residual DMSO central peak (δ_C/δ_H 39.5/2.49) was used as an internal reference. HSQC correlation peaks were assigned according to the literature,[10,11] and the quantitation of lignin units and linkages were performed as described elsewhere.[10,12] Briefly, the signals used to quantitate the relative abundances of the aromatic units and *p*-coumarates (*p*C) and tricin (T) moieties, were G_2, $S_{2,6}$, $H_{2,6}$, $pC_{2,6}$ and T_8. The volume integrals of those correlation signals involving two proton-carbon pairs ($H_{2,6}$, $S_{2,6}$ and $pC_{2,6}$) were logically halved. Lignin linkages were quantitated via the volume integrals of the A_α/A'_α, B_α, C_α, D_β and F_α correlation signals. The relative abundance of cinnamyl alcohol end-groups (I) were estimated by integration of the signals I_γ and I'_γ. The relative percentage of γ-acylation in the β−*O*−4′ substructures were determined by integrating the equivalent signals C_γ/H_γ and C'_γ/H'_γ.

Two Dimension-Heteronuclear Multiple Bond Correlation (2D-HMBC) experiment was acquired using the Bruker's pulse program "hmbcgplpndqf". The evolution time of the HMBC long-range *J*-coupling was set to 80 ms.

Quantitative ^{31}P NMR spectroscopy analysis

To perform quantitative ^{31}P NMR analysis, rye MSL was subjected to duplicate analysis using a conventional phosphitylation procedure.[13] Briefly, approximately 20 mg of moisture-free MSL, meticulously weighed, was introduced into an NMR tube and dissolved in 300 µL of a pyridine/$CDCl_3$ solution (1.6/1.0, v/v). To this mixture were added 150 µL of an internal standard solution (*N*-hydroxy-5-norbornene-2,3-dicarboximide, NHND, 9.8 mg/ml) and 75 µL of a relaxation reagent solution (chromium (III) acetylacetonate, 10.5 mg/mL), both prepared independently using the aforementioned pyridine/$CDCl_3$ solution. Afterward, 75 µL of derivatization reagent, 2-chloro-4,4,5,5-tetramethyl-1,3,2- dioxaphospholane (TMDP), were added and the tube was shaken until a homogeneous solution was formed, which was immediately analyzed. The acquisition of the ^{31}P NMR spectra was performed on a Bruker Avance NEO 500 MHz equipment using the pulse program "zgig", a relaxation delay of 5 s and 256 accumulated scans. Chemical shifts were calibrated with respect to the distinct signal arising from the hydroxylated-TMDP, which appeared at 132.2 ppm. The assignments of the signals in the ^{31}P NMR spectrum were in accordance with the literature.[13–15] The quantification of hydroxyl groups was conducted by measuring the amount of NHND added (with a purity of 97%). All solvents and reagents for the ^{31}P NMR analysis were purchased from Sigma Aldrich, except for NHND, which was acquired from Alfa Aesar. Peak integration was employed to estimate the quantities of the different OH-groups present in rye lignin, using the following formula:

$$X = \frac{V \cdot A \cdot IS \cdot P}{M \cdot N}$$

where X = mmol of OH-groups per gram of lignin (mmol/g), V = volume of internal standard added (mL), A = peak area, IS = internal standard concentration (mg/mL), P = internal standard purity (0.97), M = amount of lignin (g) and N = internal standard molecular mass (g/mol).

Gel-Permeation Chromatography (GPC). Detailed experimental conditions

For the GPC analysis, rye MSL preparations were previously acetylated and dissolved in tetrahydrofuran (THF). GPC was performed on a Shimadzu Prominence-i LC-2030 3D GPC system (Shimadzu, Kyoto, Japan) equipped with a photodiode array (PDA) detector, using the following conditions: column, PLgel 5 µm MIXED-D, 7.5 x 300 mm (Agilent Technologies, United Kingdom); THF as eluent; flow rate, 0.5 mL min^{-1}; temperature, 40 °C; sample detection, PDA response at 280 nm. The data acquisition and computation used LabSolution GPC software version 5.82 (Shimadzu). The molecular weight calibration was via polystyrene standards (Mw range from 5.8×10^{2} up to 3.24×10^{6} Da, Agilent Technologies).

Table S1. Assignments of the $^{13}C/^{1}H$ correlation signals in the 2D HSQC spectra from rye MSL (in DMSO–d_6).

Label	δ_C/δ_H (ppm)	Assignment
B_β	53.1/3.41	C_β/H_β in β–5' phenylcoumarans (**B**)
C_β	53.6/3.07	C_β/H_β in β–β' resinols (**C**)
MeO-	55.6/3.73	C/H in methoxyls
A_γ	59.4 /3.40. 3.72	C_γ/H_γ in normal (γ-OH) β–*O*–4' substructures (**A**)
F_β	59.3/2.76	C_β/H_β in spirodienones (**F**)
I_γ	61.5/4.09	C_γ/H_γ in cinnamyl alcohol end–groups (**I**)
B_γ	62.6/3.68	C_γ/H_γ in β–5' phenylcoumarans (**B**)
A'_γ	62.7/3.83. 4.30	C_γ/H_γ in γ-acylated β–*O*–4' substructures (**A'**)
$I'_{\gamma(pC)}$	63.9/4.77	C_γ/H_γ in γ-*p*-coumaroylated cinnamyl alcohol end–groups (**I'**)
$I'_{\gamma(Ac)}$	64.1/4.63	C_γ/H_γ in γ-acetylated cinnamyl alcohol end–groups (**I'**)
C_γ	71.0/3.82. 4.18	C_γ/H_γ in β–β' resinols (**C**)
A_α	71.8/4.87	C_α/H_α in β–*O*–4' substructures (**A**)
$A'_{\beta(G)}$	80.5/4.53	C_β/H_β in γ-acylated β–*O*–4' alkyl-aryl ethers (**A'**) linked to a G unit
F_α	81.2/5.02	C_α/H_α in spirodienones (**F**)
Aox_β	82.5/5.12	C_β/H_β in α-oxidized β–*O*–4' substructures (**Aox**)
$A_{\beta(H)}$	83.0/4.50	C_β/H_β in β–*O*–4' alkyl-aryl ethers (**A**) linked to a H unit
D_α	83.3/4.82	C_α/H_α in 5–5' dibenzodioxocins (**D**)
$F_{\alpha'}$	83.5/4.71	$C_{\alpha'}/H_{\alpha'}$ in spirodienones (**F**)
$A'_{\beta(S)}$	83.5/4.29	C_β/H_β in γ-acylated β–*O*–4' alkyl-aryl ethers (**A'**) linked to a S unit
$A_{\beta(G)}$	83.9/4.26	C_β/H_β in β–*O*–4' alkyl-aryl ethers (**A**) linked to a G unit
C_α	84.9/4.67	C_α/H_α in β–β' resinols (**C**)
D_β	85.4/3.86	C_β/H_β in 5–5' dibenzodioxocins (**D**)
$A_{\beta(S)}$	85.9/4.12	C_β/H_β in β–*O*–4' alkyl-aryl ethers (A) linked to a S unit
$A_{\beta(T)}$	86.2/4.36, 86.6/4.27	C_β/H_β in β–*O*–4' substructures linked to tricin (**A**)
B_α	86.9/5.47	C_α/H_α in phenylcoumarans (**B**)
T_8	94.0/6.56	C_8/H_8 in tricin (**T**)
T_6	98.7/6.22	C_6/H_6 in tricin (**T**)
$S_{2.6}$	103.8/6.69	C_2/H_2 and C_6/H_6 in etherified syringyl units (**S**)
$T_{2',6'}$	103.9/7.28	$C_{2'}/H_{2'}$ and $C_{6'}/H_{6'}$ in tricin (**T**)
T_3	104.6/7.02	C_3/H_3 in tricin (**T**)
G_2	110.9/7.00	C_2/H_2 in guaiacyl units (**G**)
FA_2	111.0/7.32	C_2/H_2 in ferulates (**FA**)
pC_8/FA_8	113.5/6.30	C_8/H_8 in *p*–coumarates (***p*C**) and ferulates (**FA**)
$H_{3,5}$	114.5/6.68	C_3/H_3 and C_5/H_5 in *p*-hydroxyphenyl units (**H**)
G_5/G_6	114.9/6.74. 6.94	C_5/H_5 and C_6/H_6 in guaiacyl units (**G**)
FA_5	113.8/6.75	C_5/H_5 in ferulates (**FA**)
$pC_{3,5}$	115.5/6.77	C_3/H_3 and C_5/H_5 in *p*-coumarates (***p*C**)
FA_6	122.9/7.13	C_6/H_6 in ferulates (**FA**)
J_8	126.3/6.76	C_8/H_8 in cinnamaldehyde end-groups (**J**)
$H_{2,6}$	127.6/7.17	C_2/H_2 and C_6/H_6 in *p*-hydroxyphenyl units (**H**)
I_β	128.2/6.21	C_β/H_β in cinnamyl alcohol end–groups (**I**)
I_α	128.4/6.44	C_α/H_α in cinnamyl alcohol end–groups (**I**)
$pC_{2,6}$	129.9/7.45	C_2/H_2 and C_6/H_6 in *p*-coumarates (***p*C**)

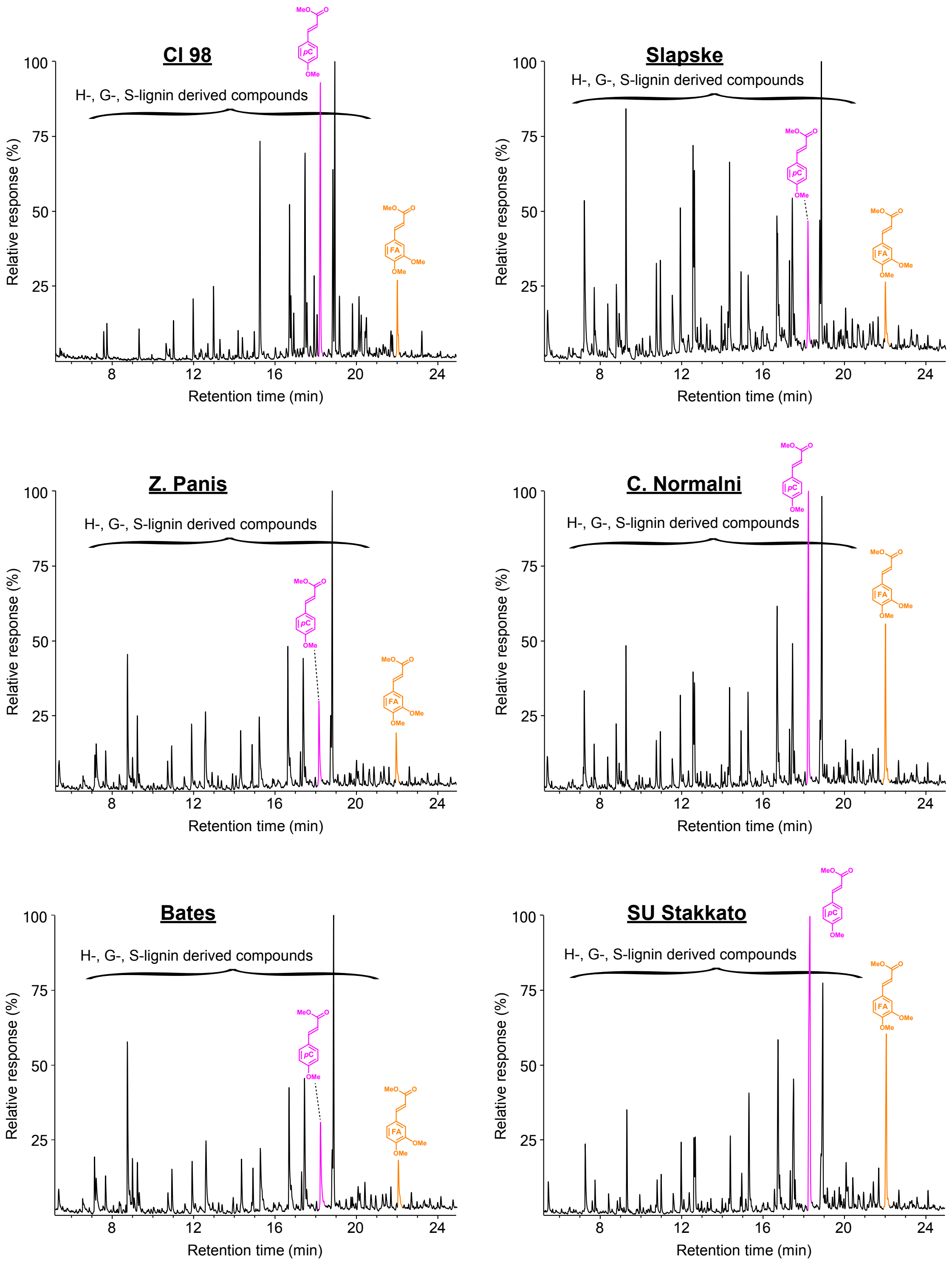

Figure S1. Py-TMAH chromatograms of the MSLs isolated from different rye varieties. *p*C is the fully methylated *p*-coumaric acid (methyl *trans*-4-*O*-methyl-*p*-coumarate) and FA is the fully methylated ferulic acid (methyl *trans*-4-*O*-methyl-ferulate).

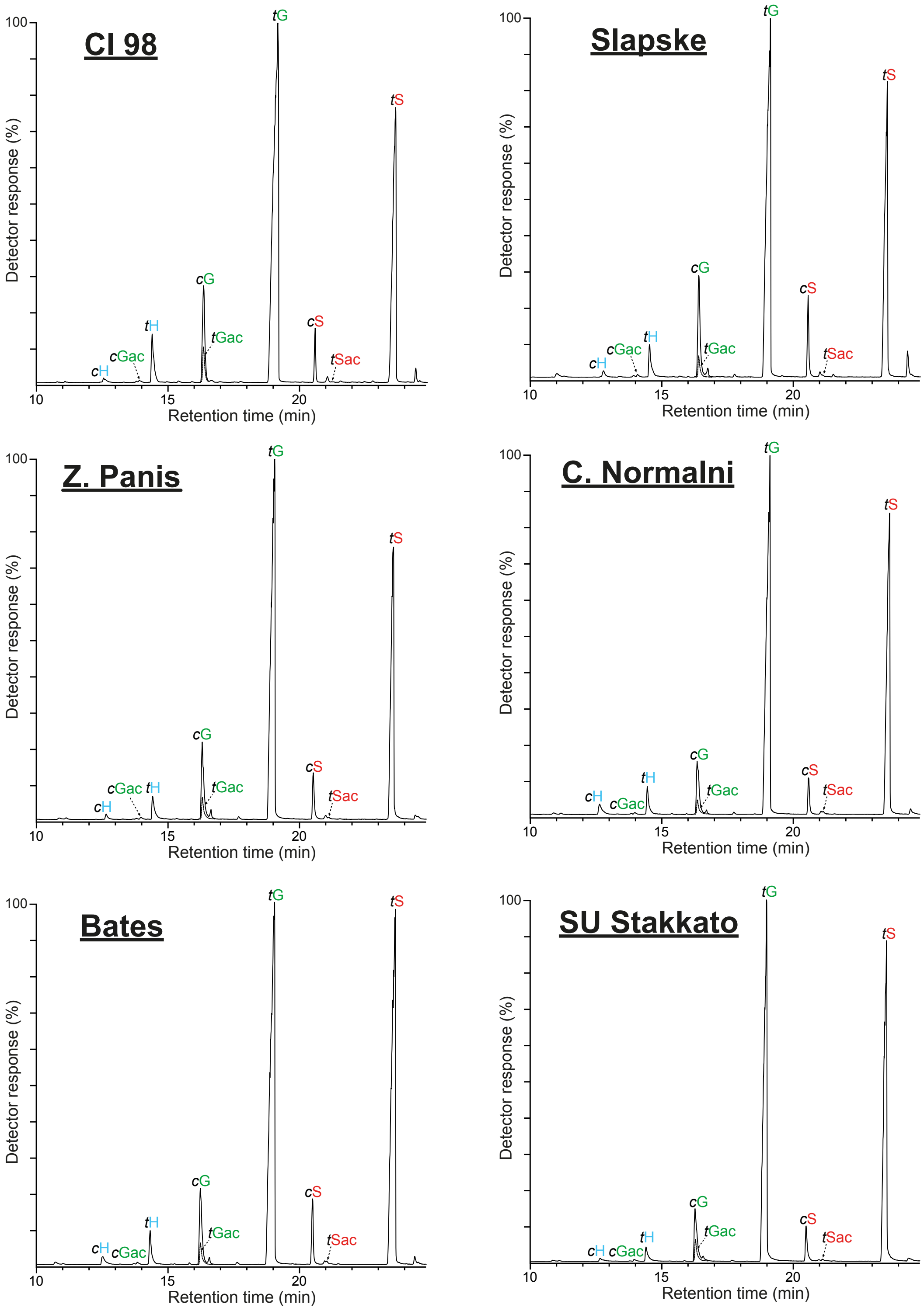

Figure S2. Reconstructed ion chromatograms (sum of the ions at *m/z* 192 + 206 + 222 + 236 + 252 + 266, which are characteristic of the γ-acetylated and γ-OH (H, G, and S lignin units) of the DFRC′ degradation products of the MSLs isolated from different rye varieties. *c*H, *t*H, *c*G, *t*G, *c*S, and *t*S are the normal (γ-OH) *cis*- and *trans*-*p*-hydroxyphenyl, guaiacyl, and syringyl monomers, respectively (as their propionylated derivatives). *t*Hac, *c*Gac, *t*Gac, *c*Sac, and *t*Sac are the originally γ-acetylated *cis*- and *trans*-*p*-hydroxyphenyl, guaiacyl, and syringyl monomers, respectively (as their propionylated derivatives).

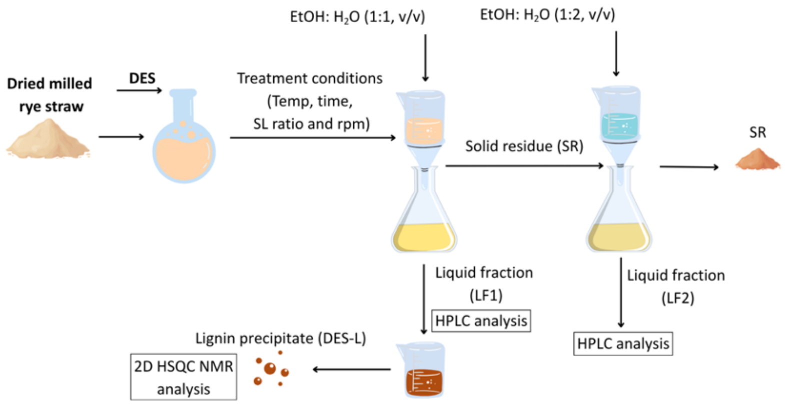

Figure S3: Flowchart illustrating the sequential steps of the deep eutectic solvent (DES) treatment used for processing rye straw samples in this study.

REFERENCES

(1) Björkman, A. Isolation of Lignin from Finely Divided Wood with Neutral Solvents. *Nature* **1954**, *174* (4440), 1057–1058. https://doi.org/10.1038/1741057a0.

(2) Ralph, J.; Hatfield, R. D. Pyrolysis-GC-MS Characterization of Forage Materials. *J. Agric. Food Chem.* **1991**, *39* (8), 1426–1437. https://doi.org/10.1021/jf00008a014.

(3) Bocchini, P.; Galletti, G. C.; Camarero, S.; Martinez, A. T. Absolute Quantitation of Lignin Pyrolysis Products Using an Internal Standard. *J. Chromatogr. A* **1997**, *773* (1–2), 227–232. https://doi.org/10.1016/S0021-9673(97)00114-3.

(4) Lu, F.; Ralph, J. Derivatization Followed by Reductive Cleavage (DFRC Method), a New Method for Lignin Analysis: Protocol for Analysis of DFRC Monomers. *J. Agric. Food Chem.* **1997**, *45* (7), 2590–2592. https://doi.org/10.1021/jf970258h.

(5) Lu, F.; Ralph, J. DFRC Method for Lignin Analysis. 1. New Method for β-Aryl Ether Cleavage: Lignin Model Studies. *J. Agric. Food Chem.* **1997**, *45* (12), 4655–4660. https://doi.org/10.1021/jf970539p.

(6) Lu, F.; Ralph, J. The DFRC Method for Lignin Analysis. 2. Monomers from Isolated Lignins. *J. Agric. Food Chem.* **1998**, *46* (2), 547–552. https://doi.org/10.1021/jf970676m.

(7) del Río, J. C.; Prinsen, P.; Rencoret, J.; Nieto, L.; Jiménez-Barbero, J.; Ralph, J.; Martínez, Á. T.; Gutiérrez, A. Structural Characterization of the Lignin in the Cortex and Pith of Elephant Grass (*Pennisetum Purpureum*) Stems. *J. Agric. Food Chem.* **2012**, *60* (14), 3619–3634. https://doi.org/10.1021/jf300099g.

(8) Ralph, J.; Lu, F. The DFRC Method for Lignin Analysis. 6. A Simple Modification for Identifying Natural Acetates on Lignins. *J. Agric. Food Chem.* **1998**, *46* (11), 4616–4619. https://doi.org/10.1021/jf980680d.

(9) del Río, J. C.; Marques, G.; Rencoret, J.; Martínez, Á. T.; Gutiérrez, A. Occurrence of Naturally Acetylated Lignin Units. *J. Agric. Food Chem.* **2007**, *55* (14), 5461–5468. https://doi.org/10.1021/jf0705264.

(10) del Río, J. C.; Rencoret, J.; Prinsen, P.; Martínez, A. T.; Ralph, J.; Gutiérrez, A. Structural Characterization of Wheat Straw Lignin as Revealed by Analytical Pyrolysis, 2D-NMR, and Reductive Cleavage Methods. *J. Agric. Food Chem.* **2012**, *60* (23), 5922–5935. https://doi.org/10.1021/jf301002n.

(11) Rencoret, J.; Marques, G.; Rosado, M. J.; Benito, J.; Barro, F.; Gutiérrez, A.; del Río, J. C. Variations in the Composition and Structure of the Lignins of Oat (*Avena Sativa* L.) Straws According to Variety and Planting Season. *Int. J. Biol. Macromol.* **2023**, *242*, 124811. https://doi.org/10.1016/j.ijbiomac.2023.124811.

(12) Rencoret, J.; Kim, H.; Evaristo, A. B.; Gutiérrez, A.; Ralph, J.; del Río, J. C. Variability in Lignin Composition and Structure in Cell Walls of Different Parts of Macaúba (*Acrocomia Aculeata*) Palm Fruit. *J. Agric. Food Chem.* **2018**, *66* (1), 138–153. https://doi.org/10.1021/acs.jafc.7b04638.

(13) Meng, X.; Crestini, C.; Ben, H.; Hao, N.; Pu, Y.; Ragauskas, A. J.; Argyropoulos, D. S. Determination of Hydroxyl Groups in Biorefinery Resources via Quantitative ^{31}P NMR Spectroscopy. *Nat. Protoc.* **2019**, *14* (9), 2627–2647. https://doi.org/10.1038/s41596-019-0191-1.

(14) Li, M.; Pu, Y.; Tschaplinski, T. J.; Ragauskas, A. J. ^{31}P NMR Characterization of Tricin and Its Structurally Similar Flavonoids. *ChemistrySelect* **2017**, *2* (12), 3557–3561. https://doi.org/10.1002/slct.201700735.

(15) Li, M.; Pu, Y.; Meng, X.; Chen, F.; Dixon, R. A.; Ragauskas, A. J. Strikingly High Amount of Tricin-Lignin Observed from Vanilla (*Vanilla Planifolia*) Aerial Roots. *Green Chem.* **2022**, *24* (1), 259–270. https://doi.org/10.1039/D1GC03625D.
